# Developmental dyscalculia is not associated with atypical brain activation: A univariate fMRI study of arithmetic, magnitude processing, and visuospatial working memory

**DOI:** 10.1002/hbm.26495

**Published:** 2023-11-01

**Authors:** Fu Yu Kwok, Eric D. Wilkey, Lien Peters, Ellyn Khiu, Rebecca Bull, Kerry Lee, Daniel Ansari

**Affiliations:** ^1^ Centre for Research in Child Development, National Institute of Education Nanyang Technological University Singapore; ^2^ Macquarie School of Education Macquarie University Sydney New South Wales Australia; ^3^ Brain and Mind Institute Western University London Ontario Canada; ^4^ Vanderbilt Brain Institute Vanderbilt University Nashville Tennessee USA; ^5^ Department of Psychology & Human Development Peabody College, Vanderbilt University Nashville Tennessee USA; ^6^ Department of Experimental Clinical and Health Psychology Research in Developmental Disorders Lab Ghent University Ghent Belgium; ^7^ Department of Early Childhood Education The Education University of Hong Kong Hong Kong

**Keywords:** arithmetic, Bayesian, developmental dyscalculia, fMRI, math learning difficulties, math learning disability, number processing, visuo‐spatial working memory

## Abstract

Functional neuroimaging serves as a tool to better understand the cerebral correlates of atypical behaviors, such as learning difficulties. While significant advances have been made in characterizing the neural correlates of reading difficulties (developmental dyslexia), comparatively little is known about the neurobiological correlates of mathematical learning difficulties, such as developmental dyscalculia (DD). Furthermore, the available neuroimaging studies of DD are characterized by small sample sizes and variable inclusion criteria, which make it problematic to compare across studies. In addition, studies to date have focused on identifying single deficits in neuronal processing among children with DD (e.g., mental arithmetic), rather than probing differences in brain function across different processing domains that are known to be affected in children with DD. Here, we seek to address the limitations of prior investigations. Specifically, we used functional magnetic resonance imaging (fMRI) to probe brain differences between children with and without persistent DD; 68 children (8‐10 years old, 30 with DD) participated in an fMRI study designed to investigate group differences in the functional neuroanatomy associated with commonly reported behavioral deficits in children with DD: basic number processing, mental arithmetic and visuo‐spatial working memory (VSWM). Behavioral data revealed that children with DD were less accurate than their typically achieving (TA) peers for the basic number processing and arithmetic tasks. No behavioral differences were found for the tasks measuring VSWM. A pre‐registered, whole‐brain, voxelwise univariate analysis of the fMRI data from the entire sample of children (DD and TA) revealed areas commonly associated with the three tasks (basic number processing, mental arithmetic, and VSWM). However, the examination of differences in brain activation between children with and without DD revealed no consistent group differences in brain activation. In view of these null results, we ran exploratory, Bayesian analyses on the data to quantify the amount of evidence for no group differences. This analysis provides supporting evidence for no group differences across all three tasks. We present the largest fMRI study comparing children with and without persistent DD to date. We found no group differences in brain activation using univariate, frequentist analyses. Moreover, Bayesian analyses revealed evidence for the null hypothesis of no group differences. These findings contradict previous literature and reveal the need to investigate the neural basis of DD using multivariate and network‐based approaches to brain imaging.

## Graphical abstract

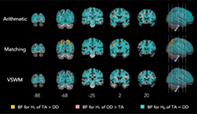

## 1 INTRODUCTION

Specific learning disorders are neurodevelopmental disorders that affect children's learning of key foundational skills in the domains of writing, reading and math. Neuroimaging has the potential to provide otherwise unobservable insights into the neurobiological correlates of behavioral deficits in specific learning disorders. For example, functional magnetic resonance imaging (fMRI) studies into the neurobiology of dyslexia (a specific learning disorder in the domain of reading) have identified that children who go on to receive a diagnosis of dyslexia exhibit an altered pattern of brain sensitivity to print at the very outset of learning to read (Yamada et al., 2011). Though no consensus has emerged, longitudinal studies have begun to tease apart the evidence for altered versus delayed neural development of dyslexia (Chyl et al., 2021) and studies have begun to demarcate possible biomarkers for response to intervention (Aboud et al., 2018).

Like dyslexia, math learning difficulties, often referred to as developmental dyscalculia (DD), is a specific learning disorder characterized by persistent and severe difficulties in an academic skill not accounted for by an intellectual disability or another mental/neurological disorder and despite access to appropriate educational support. However, though DD has a similar prevalence rate as dyslexia (Morsanyi, van Bers, McCormack, & McGourty, 2018; Shalev et al., 2000), research on the neurobiological correlates of DD is comparatively scant. There is currently no consensus surrounding the basic neurobiological correlates of severe difficulties learning math (Bugden & Ansari, 2014).

Neuroimaging studies of DD have mostly focused on a series of core deficits associated with cognitive profiles identified through behavioral studies. For example, children and adults with DD often have difficulty with basic numerical skills like processing numerical quantities (Dehaene, 2011). This deficit in number processing has been demonstrated through tasks in which children choose the larger of two symbolic numbers (e.g., Arabic digits; Bugden et al., 2021; Rousselle & Noël, 2007) or nonsymbolic quantities (e.g., arrays of dots; Mazzocco et al., 2011; Wilkey et al., 2020), map between symbolic and nonsymbolic quantities (Noël & Rousselle, 2011), rapidly respond to whether or not a series of digits is in numerical order (Morsanyi, van Bers, O'Connor, & McCormack, 2018), and accurately place a number on a number line (Schneider et al., 2018). Accordingly, researchers have sought to identify the neural correlates of basic nonsymbolic and symbolic number processing and to evaluate their neural correlates in groups of DD participants. To date, findings are mixed, with some studies showing that DD children have increased activation in parietal regions known to process numerical magnitudes (nonsymbolic; Kaufmann et al., 2011; Kaufmann, Vogel, Starke, Kremser, & Schocke, 2009 and symbolic; McCaskey et al., 2018), others reporting decreased activation in the same regions (symbolic; Mussolin et al., 2010; and nonsymbolic Price et al., 2007), and still others reporting no group difference anywhere in the parietal lobe (nonsymbolic; Kucian et al., 2006, 2011; McCaskey et al., 2017).

Two recent meta‐analyses provide two common findings across this literature. Both Martinez‐Lincoln et al. (2023) and Tablante et al. (Tablante et al., 2023) report that a region of the right anterior intraparietal sulcus (IPS) is consistently less active for individuals with math difficulties than for their typically achieving (TA) peers and that one region in the right insula is more active for the math difficulty group. However, there is less specificity about what elicits these differences in processing. Tasks contributing to the IPS cluster include the processing of math facts, magnitude comparison, ordinality, color comparison, spatial working memory, and transitive reasoning. The cluster reported in Tablante et al. even includes voxels contributed from four analyses of differences in brain structure. While the right IPS is consistently associated with magnitude processing across studies of mathematical cognition (Sokolowski et al., 2017), it also frequently associated with attentional allocation (Connolly et al., 2016), visuospatial working memory (VSWM) (Klingberg, 2006; Silk et al., 2010), control of grasping and reaching (Grefkes & Fink, 2005), and task difficulty contrasts generally (Bankó et al., 2011; Bokde et al., 2005; Gould et al., 2003). Tasks contributing to the insula cluster include ordinality and processing math facts. However, this region is also associated with a very broad array of processing, ranging from sensory and affective processing to high‐level, domain‐general cognition (Uddin et al., 2017). Therefore, while there are at least two consistent differences in brain function anatomically when comparing DD and TA, the developmental mechanisms that characterize this functionally remain obscure.

Behaviorally, it is well established that children with DD experience deficits in the encoding and recollection of arithmetic facts, which often manifests as poor arithmetic fluency (Geary, 1993; Geary et al., 2012). Thus far, several imaging studies have indicated that children with DD exhibit reduced activation in superior parietal structures and the ventral occipito‐temporal cortex during arithmetic problem solving (Ashkenazi et al., 2012; Berteletti et al., 2014; Peters et al., 2018). However, it has also been reported that DD children have increased activation in these same regions during similar tasks, involving both addition and subtraction (Rosenberg‐Lee et al., 2015). Here again, at present, our ability to distill a consistent pattern of data linking poor arithmetic fluency in DD to neurobiological mechanisms is limited.

A third frequently identified behavioral marker of DD is reduced VSWM capacity (Mammarella et al., 2015, 2018; Szűcs, 2016). Low working memory capacity has been linked to decreased efficiency of learning to transcode numbers across nonsymbolic and symbolic formats (Camos, 2008). More generally, working memory is important for arithmetic, which involves manipulating numbers and holding relevant information in mind during problem solving. Neuroimaging of TA populations has shown that parietal activation during a VSWM task predicted arithmetic 2 years later (Dumontheil & Klingberg, 2012), and conversely, that fronto–parietal activity during an arithmetic task correlated with VSWM ability (Ashkenazi et al., 2013; Metcalfe et al., 2013). Only one study to date has investigated brain activity of DD children during a VSWM task. Using a dot‐matrix task (adapted from the Corsi block‐tapping task; Dumontheil & Klingberg, 2012), Rotzer et al. compared a group of 10 DD children to a group of 11 control children, aged 8–10 years old (Rotzer et al., 2009). Compared to controls, the DD group showed weaker activation in the right IPS, right insula, and right inferior frontal lobe. These inferior frontal findings stand in contrast to the two meta‐analyses referenced above, which report increased activation of the right insula and inferior frontal lobe for children with math difficulties during math and ordinality tasks. Given this limited evidence, more work is needed to understand potential differences in the neural correlates of VSWM for children with DD.

In reviewing the literature on functional neuroimaging of DD, we identify three main limitations that have hindered prior efforts to make strong inferences about the neurocognitive basis of DD. First, the sample size of fMRI studies on children with DD tends to be small. Low statistical power results in false positives and inflated effect sizes, which both lead to replication failures (Button et al., 2013). While earlier neuroimaging studies have been able to connect DD with particular brain activation profiles, more recent studies have often not succeeded in replicating these findings, calling into question the reliability of the original findings. Second, there is a lack of consensus over the operational definition of DD across neuroimaging studies. The fMRI studies of DD adopt a mixture of clinical diagnosis and math achievement cut‐offs that vary widely from study to study (see Table 1; Peters & Ansari, 2019). Most study samples are not well‐aligned with the DSM‐V‐TR criteria and do not track math skills longitudinally. A third critical limitation of the extant research is the lack of studies that have investigated multiple candidate mechanisms for neural differences between DD and TA children within the same sample (e.g., comparing the neural correlates of both arithmetic and VSWM in the same groups of children). The same set of fronto–parietal mechanisms are involved in various cognitive functions frequently associated with mathematical tasks, including the processing of symbolic and nonsymbolic numerical magnitudes, executive functions (i.e., working memory, attentional allocation, and inhibition), and arithmetic fact retrieval. Therefore, any number of theoretical explanations involving these mechanisms could account for the fronto–parietal abnormalities so often observed in DD populations. To disambiguate one theory from another, it is imperative to image multiple tasks in the same sample of children with DD. Table 1 details the sample size, age range, criteria used for DD designation, tasks imaged, and minimum and maximum effect size of significant differences in neural activity observed between DD and TA control groups for all published fMRI studies of DD to date in pediatric populations, to the best of our knowledge. All three of these limitations can be readily observed in Table 1, including in previous studies conducted by the current study's authors.

**TABLE 1:** Details of pediatric fMRI studies comparing children with math learning difficulties and their typically achieving peers.

First author	Year	DD *n*	TA *n*	DD characterization	Age range	Task	Group difference effect size range (Cohen's *D*)
Ashkenazi et al. (2012)	2012	17	17	<25th pct	7–9	Addition	0.92–1.18
Ashkenazi	2013	17	17	<27th pct	7–9	Addition	0.96–1.41
Berteletti et al. (2014)	2014	20	20	<16th pct	8–13	Multiplication	0.68*–1.10
Davis et al. (2009)	2009	24	24	<25th pct	8–9	Exact and approximate addition	1.73–2.36
De Smedt et al. (2011)	2011	8	10	<16th pct	10–12	Addition	2.71–2.87
Dinkel et al. (2013)	2013	16	16	<10th pct	6–10	Non‐symbolic comparison and addition	Not reported
Iuculano et al. (2015)	2015	15	15	<16th pct	7–9	Addition	0.83–1.87
Kaufmann, Vogel, Starke, Kremser, Schocke, & Wood (2009)	2009	9	9	<16th pct	8–10	Non‐symbolic comparison and spatial Judgment	2.75–3.00
Kaufmann, Vogel, Starke, Kremser, & Schocke (2009)	2009	6	6	<16th pct	8–12	Ordinality	4.00–4.43
Kovas et al. (2009)	2009	13	13	<7th pct	10	Non‐symbolic comparison	Not reported
Kucian et al. (2006)	2006	18	20	Clinical diagnosis	10–12	Exact and approximate addition Magnitude comparison	1.29**–1.40**
Kucian et al. (2011)	2011	15	15	Clinical diagnosis	9–12	Non‐symbolic comparison	1.10–1.69
Kucian	2011	16	16	Clinical diagnosis	8–10	Ordinality	0.93–1.44
Michels	2018	15	16	<10th pct	7–11	Ordinality	1.19–1.50
McCaskey et al. (2017)	2017	16	14	Max. score of 67 out of 83	11–16	Non‐symbolic comparison, spatial judgment, and mental rotation	1.56–1.64
McCaskey et al. (2018)	2018	14	11	<10th pct	8–11	Ordinality	1.33–2.18
Mussolin et al. (2010)	2010	15	15	Clinical referral	9–11	Symbolic comparison	1.29–1.54
Peters et al. (2018)	2018	16	22	Clinical diagnosis	9–12	Subtraction	Not reported
Price et al. (2007)	2007	8	8	<7th pct	11–12	Non‐symbolic comparison	2.94–3.47
Rosenberg‐Lee et al. (2015)	2015	16	20	<25th pct	7–9	Addition	0.94–1.40
Rotzer et al. (2009)	2009	10	11	<7th pct	8–10	Corsi block tapping	1.58**–1.92**
Schwartz	2018	16	18	25th pct	8–12	Transitive reasoning	1.49

*Note*: Effect Size calculation details and code are available on the OSF project page linked in the text.

Abbreviations: DD, developmental dyscalculia; TA, typically achieving.

* Reported at *p* = .09;

** Not significant after correcting for multiple comparisons (uncorrected *p* < .001).

The current fMRI study investigating the neurobiological correlates of DD seeks to address the aforementioned limitations, while employing a similar univariate fMRI analytic approach as most previous studies of DD and making the following methodological improvements. First, the sample recruited for the current study is the largest collection of DD children imaged to date. Second, stringent criteria were used for defining the DD sample. We only included children who either performed in the bottom 10th percentile of the test of early mathematics ability‐3 (TEMA‐3) within the broader project sample across kindergarten and first grade (Ng et al., 2014; Ng & O'Brien, 2020) or who were identified via a school entry screening tool as poor performers in mathematics who required learning support. Lastly, the current study design employs three fMRI tasks across the same set of participants to tap into the key mechanisms that have been suggested as the neural correlates of DD (i.e., arithmetic, number matching, and a dot‐matrix task). With a set of carefully pre‐registered analyses (https://osf.io/vsr8b), publicly available data, and a complementary set of Bayesian post‐hoc tests, the current study seeks to make progress toward developing a replicable consensus in the field.

## 2 METHODS

### 2.1 Participants

All participants were invited based on previous inclusion in a larger longitudinal study (Ng et al., 2014; Ng & O'Brien, 2020), where cognitive data were obtained across four timepoints over 3 years (Kindergarten 1, Kindergarten 2, and Grade 1). Seventy‐seven Grade 3 children with no prior history of neurological or psychiatric conditions enrolled in the study (for recruitment method, see Data S1). Of these children, one was excluded due to medical reasons and eight did not complete scanning successfully, making the total sample size 68 children (mean age = 8.95 years, SD = 0.34; 30 male). Parental informed consent and child assent were obtained and children completed a pediatric MRI protocol. Ethics approval was received from the Nanyang Technological University Institutional Review Board.

### 2.2 Group categorization

Participants were categorized into two groups prior to the Grade 3 timepoint. The DD group included 30 children who either (a) participated in a learning support for math (LSM) intervention program (i.e., children who were identified by the Ministry of Education in Singapore as having difficulty in mathematics, through a confidential screener taken by all children when they enter primary school), or (b) scored at the bottom 10th percentile of a standardized math test (i.e., TEMA; see Data S1) in Grade 1, but were not identified by the screener as having math difficulties. These two groups did not differ from each other at any timepoint on the TEMA or on any numerical assessment at Grade 3 (see Data S1 for details). While there was no formal clinical assessment, including no general IQ measure, we believe these children meet the criteria for Specific Learning Impairment in mathematics (i.e., DD) due to the following reasons: (i) persistent difficulties for at least 6 months, despite the provision of targeted interventions, and (ii) the learning difficulties were not better accounted for by intellectual disabilities, uncorrected visual or auditory acuity, other mental or neurological disorders, lack of language proficiency, or inadequate educational instruction.

The 38 TA controls scored above the 25th percentile of the TEMA when they were assessed in Grade 1. Additionally, TA children were matched on the following, with the first two criteria being prioritized: school (i.e., same primary school as the DD child and if not possible, the same kindergarten in K2), age (i.e., within 3 months of age from the DD child), gender, race, ethnicity, and socio‐economic status. Latent profile analysis was conducted to ensure the stability of the grouping at Grade 3 (see Data S1 for more justification on LSM, for other low achieving children, and for a stability analysis of the groups over time).

### 2.3 Experimental tasks

#### 2.3.1 Arithmetic

**FIGURE 1:**
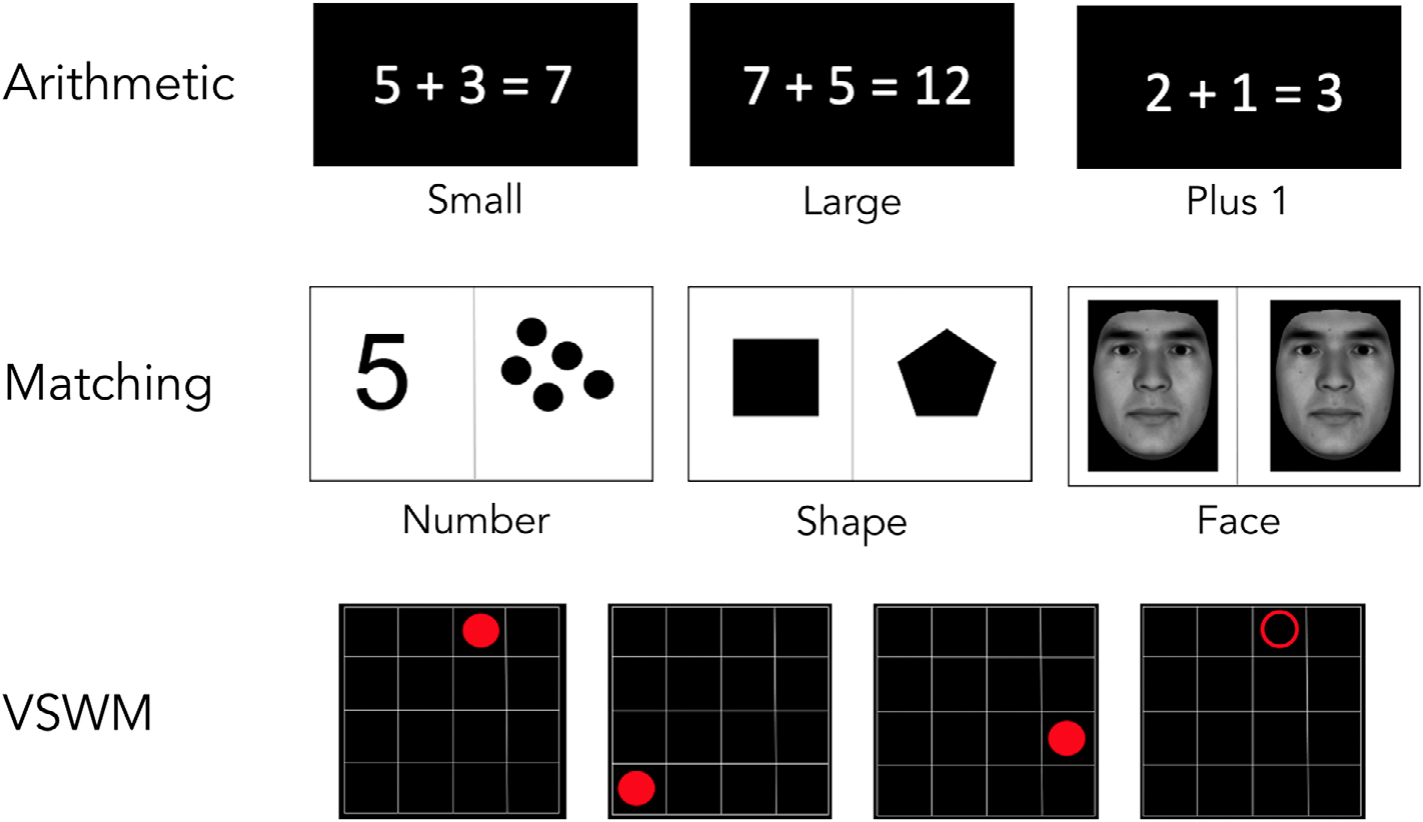
Example stimuli per task. Top panel (left‐to‐right): arithmetic task conditions [solution] = small [incorrect], large [correct], and plus 1 [correct]. Middle panel (left‐to‐right): matching task conditions [solution] = number [same], shape [different], and face [same]). Bottom panel (stimuli were presented sequentially, represented here as progressing left‐to‐right): VSWM (3‐span experimental trial displayed; participants would respond that the circle had passed through the location of the empty circle. Control condition trials differed in that the color of the dots being displayed was blue instead of red, and because participants were asked to press a button when the open circle appeared regardless of its location).

To investigate arithmetic problem solving, participants completed two runs of a single‐digit addition verification task (Matejko & Ansari, 2017). They were presented with a problem and a solution and were instructed to evaluate if that solution was correct (50% of trials). All stimuli were shown in white on a black background (see Figure 1, top panel). The task comprised three conditions: small problems (solution smaller than or equal to 10), large problems (solution >10), and plus1 problems (trial list available in Data S1). Tie problems and problems containing zero as operand were excluded from the trial list. Each run consisted of 36 problems (12 per condition).

#### 2.3.2 Matching

A matching task was used to assess basic number processing, used for processing the primary semantic representation of both symbolic and non‐symbolic numerical magnitudes (Emerson & Cantlon, 2012, 2015; Skagenholt et al., 2018). In the number condition, participants were simultaneously presented with a number symbol and a set of dots and were asked to decide whether both stimuli represented the same quantity (50% of trials). In the shape condition, two shapes were presented (e.g., circle and star), and participants were asked to determine if they were the same (50% of trials) or different. A third condition, face matching, was included. This task was matched in difficulty to the number condition, as piloting of the task showed that the number and shape conditions differed in difficulty level. Participants were presented with two front‐facing Asian faces (created using FaceGen Artist, https://facegen.com/index.htm) and were asked to determine if those two faces represented the same identity (50% of trials; trial list available in Data S1). Participants completed two runs, and each run comprised two blocks of six trials per condition (36 trials total). A cue with an example stimulus (see Figure 1, middle panel) preceded each block.

#### 2.3.3 Visuo‐spatial working memory

A task adapted from Dumontheil and Klingberg (2012) was employed to investigate working memory. In the experimental condition, participants observed a red dot move through a 4 × 4 white grid on a black background. After the red dot disappeared, an empty red circle appeared and participants were asked to respond with a button press—right if its location matched one of the previous locations the red dot had passed through and left if it did not match (50% of trials; see Figure 1, bottom panel). In the control condition, participants watched a blue dot move through the grid. When the target stimulus appeared (i.e., an empty blue circle), participants were instructed to respond by pressing the button on their right hand regardless of the location of target stimulus. Each condition comprised low load (dot passed through three locations in the grid) and high load trials (five grid locations). Participants completed two runs, with each run consisting of 6 trials for both loads in both conditions.

All tasks were presented as block designs and had an initial fixation block (6500 ms) and an end fixation block (12,000 ms). Each block consisted of six trials of a condition, with a jittered inter‐trial interval (ITI) averaged at 1500 ms. In the arithmetic task, each problem was presented for 4500 ms and in the matching task for 2000 ms. In the VSWM task, the duration of a trial depended on the load. Each dot location was presented for 500 ms followed by a blank grid of 500 ms. After the dot passed through the grid, a wait screen appeared (1500 ms) followed by the target screen (1500 ms). For all tasks, participants were asked to respond as quickly and as accurately as possible, and to respond even after the stimulus had disappeared; responses and response times were also recorded during the ITI. Every participant completed all trials, in randomized order. Latin square counterbalancing of the conditions was used to minimize order effects. Interblock intervals lasted on average 9 s (i.e., 6, 9, or 12 s).

All tasks were presented using E‐Prime 2.0 (Psychology Software Tools, Pittsburg, PA), and participants responded by pressing a button on a response box. Stimuli were projected onto a screen at the end of the scanner bore visible through a mirror mounted on the head coil. For each task, participants whose accuracy was <50% across conditions and runs were excluded from analyses, task‐wise. Participants with 0% accuracy on a condition were excluded from analyses of that task. These criteria led to the exclusion of three children in arithmetic (one TA and two DD), four in VSWM (one TA and three DD), and none in matching.

### 2.4 MRI acquisition

Images were acquired on a Siemens 3T MAGNETOM Prisma MRI scanner (Siemens Prisma, Erlangen, Germany), equipped with a 32‐channel head coil. We used an MPRAGE sequence for a high resolution T1‐weighted imaged: TR = 2300 ms; TE = 2.29 ms; FOV = 204 mm; voxel size of 0.9 × 0.9 × 0.9 mm^3^, ascending, and slice thickness = 0.94 mm. For functional data, the T2* weighted EPI images were obtained using the oblique axial plane: TR = 1000 ms; TE = 28 ms; 48 interleaved slices; flip angle = 40°; FOV = 208 mm; in‐plane voxel size of 2.5 × 2.5 × 2.5 mm and a slice thickness of 2.5 mm.

### 2.5 MRI preprocessing and analysis

The open‐source BIDS application fMRIPrep 1.4.1 (Esteban et al., 2019) was used to preprocess all data. A full description of the preprocessing pipeline details can be found in Data S1. In short, structural images were corrected for inhomogeneities and normalized to standardized MNI space (MNI‐ICBM 152). Functional images were slice‐time corrected, head‐motion parameters were estimated, and the images were co‐registered to the T1w reference. The BOLD time‐series were normalized to standardized MNI space and spatially smoothed with a 6 mm fwhm Gaussian kernel. MRI data were analyzed in SPM12 using a general linear model. A two‐gamma hemodynamic response function was used to model the expected BOLD signal for each trial per condition (correct trials only).

The analysis plan was preregistered on the Open Science Framework (https://osf.io/vsr8b and https://osf.io/dz7fx). One contrast of interest was specified for each task. For the arithmetic task, this was [(Large + Small) > Plus1], as it contrasts problems in which participants were required (or not required) to calculate (the Plus1 condition can be solved by counting). For the matching task, the contrast [Number > Shape] was the main contrast of interest, as it taps into the matching of a number with its quantity, while controlling for processes of no interest such as visual perception and decision making. The contrast [Number > Face] was used for a difficulty matched, follow‐up analysis, as pre‐registered. Finally, for VSWM, the contrast [VSWM (collapsed over load) > Control (collapsed over load)] was estimated, to isolate activity related to holding visuo‐spatial information in working memory. For each task, a whole‐brain, within‐subjects *t‐*test including all participants was run. To model group differences (TA vs. DD) in task‐related neural activity, a whole‐brain independent samples *t*‐tests was run for each task. An initial uncorrected threshold of *p* < .001 and a cluster level correction threshold of *p* < .05 was applied across all analyses using the REST AlphaSim algorithm (“‐acf” flag) in AFNI to estimate noise.

## 3 RESULTS

### 3.1 FMRI task behaviors

**FIGURE 2:**
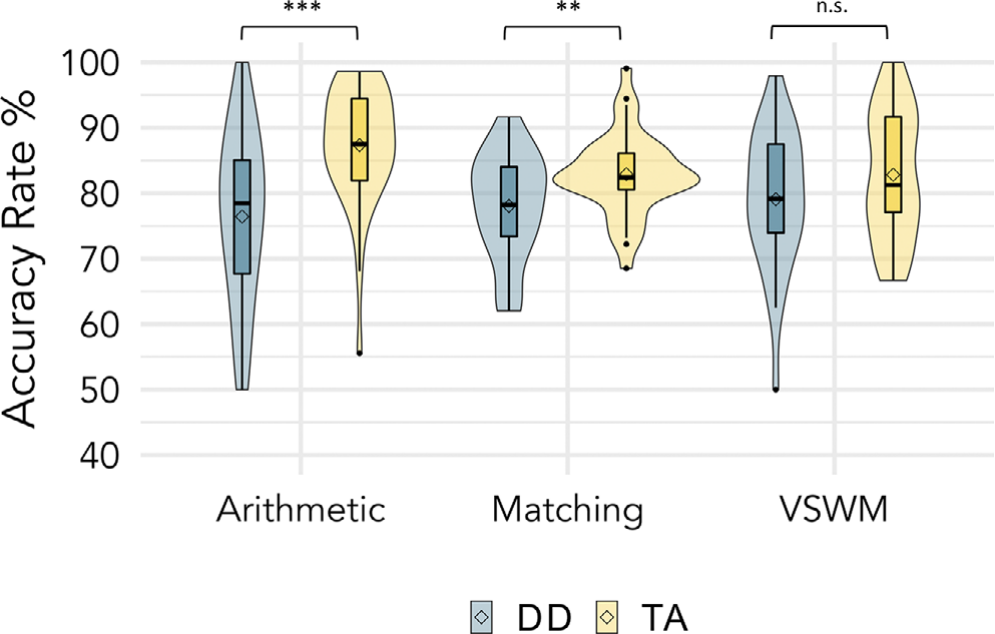
fMRI task accuracy rates grouped by math achievement group. DD (blue), developmental dyscalculia; TA (yellow), typically achieving. Box plot hinges represent 25th and 75th percentile of distributions, whiskers extend from hinge to the largest value not beyond 1.5 times the interquartile range, the middle solid line represents the median value, and the diamond represents the mean. *** *p* < .001, ** *p* < .01.

On average, children with DD were less accurate than TA children in the arithmetic task [*t*(63) = 3.96, *p* < .001, Cohen's *d* = 0.99], and the matching task [*t*(65) = 2.89, *p* = .005, Cohen's *d* = 0.71], but did not differ significantly in the VSWM task [*t*(66) = 1.78, *p* = .079, Cohen's *d* = 0.44] (Figure 2). Accuracy rates and response times by group, task, and condition are presented in Table 2. Behaviors of fMRI task performance that mirrored the pre‐registered fMRI contrasts were also analyzed by combining accuracy and response time into inverse efficiency scores (IES = response time/accuracy rate) and then analyzed with a 2 × 2 repeated measures ANOVA. For the arithmetic task, the group × condition interaction [Group (DD, TA) × Condition (Large and Small, Plus1)] was significant [*F*(1) = 16.35, *p* < .001, *η* ^2^ = 0.033], indicating the DD group had a greater behavioral difference between task conditions than the TA group. For the Matching task, the group × condition interaction [Group (DD, TA) × Condition (Number, Shape)] was not significant [*F*(1) = 1.23, *p* = .272, *η* ^*2*^ = 0.025], which suggested the degree of difference between the number and shape conditions did not differ significantly between groups. Lastly, For the VSWM task, the group × condition interaction [Group (DD, TA) × Condition (3‐Span and 5‐Span, 3‐Span Control, and 5‐Span Control)] was significant [*F*(1) = 8.08, *p* = .006, *η* ^*2*^ = 0.025], indicating the DD group had a greater behavioral difference between task conditions than the TA group.

**TABLE 2:** Descriptive statistics for fMRI tasks by math achievement group and condition.

				Accuracy %			Response time (ms)		
	Group	Condition	*n*	*M* (SD)	Min	Max	*M* (SD)	Min	Max
Arithmetic	TA	Large	37	79.2 (13.5)	29.2	100	2997 (617)	1002	4100
	DD	Large	28	61.5 (18.0)	50.0	100	3206 (907)	971	4793
	TA	Small	37	89.1 (11.3)	45.8	100	2440 (545)	993	3512
	DD	Small	28	77.4 (15.6)	41.7	100	3036 (710)	1503	4642
	TA	Plus1	37	93.8 (8.9)	58.3	100	2098 (484)	966	3694
	DD	Plus1	28	90.5 (11.4)	58.3	100	2346 (678)	1431	4566
Matching	TA	Numbers	37	77.3 (9.7)	52.8	100	1507 (179)	1188	1983
	DD	Numbers	30	74.0 (9.5)	55.6	91.7	1619 (220)	1234	2046
	TA	Shapes	37	92.8 (6.2)	72.2	100	1263 (180)	835	1668
	DD	Shapes	30	89.0 (7.3)	75.0	100	1364 (239)	1013	2139
	TA	Faces	37	78.7 (8.9)	55.6	97.2	1548 (173)	1131	1891
	DD	Faces	30	71.2 (12.4)	47.2	91.7	1658 (237)	1340	2501
Visuo‐spatial working memory	TA	Span 3	37	77.0 (18.5)	41.7	100	1273 (179)	937	1640
	DD	Span 3	27	67.9 (20.8)	33.3	100	1335 (327)	746	2443
	TA	Span 5	37	73.4 (15.2)	41.7	100	1277 (188)	927	1785
	DD	Span 5	27	66.0 (20.3)	33.3	100	1379 (298)	890	2266

*Note*: Response times are calculated for correct trials only.

Abbreviations: DD, developmental dyscalculia; TA, typically achieving.

### 3.2 Pre‐registered frequentist approach

#### 3.2.1 Whole‐group task contrasts

**FIGURE 3:**
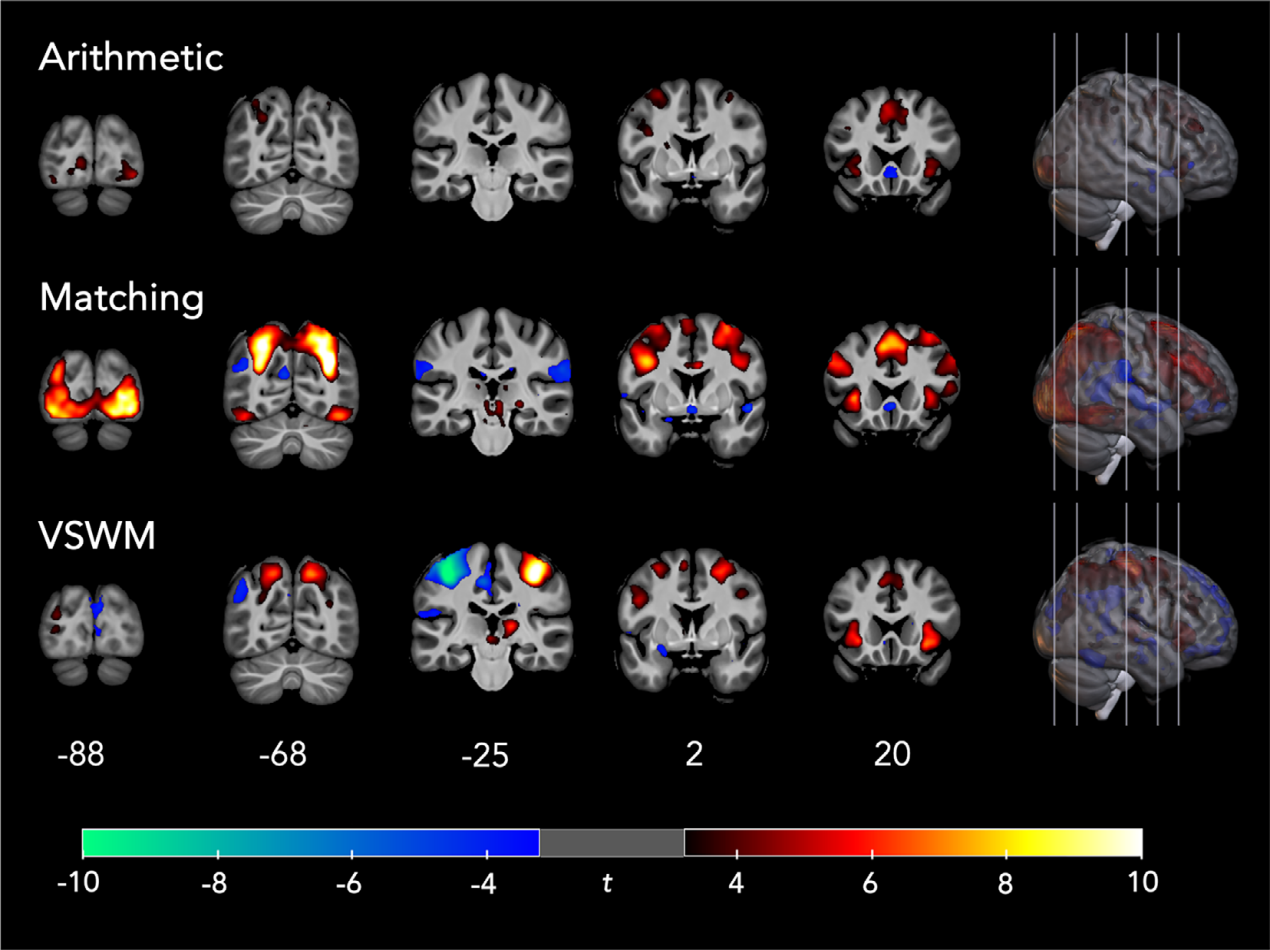
Maps of activation for the within‐subjects *t*‐tests for all three tasks, main contrasts of interest. Significance threshold was *t* = 3.22 at *p* < .001 uncorrected, cluster‐corrected at *k* = 29 for all comparisons.

Whole‐brain within‐subjects *t‐*tests across all participants (TA + DD) were run for each of the three tasks (see Figure 3). A table with information on cluster sizes, *t‐*values and peak coordinates for each task can be found in Table 3 (see Table S1 for further details).

**TABLE 3:** Significant clusters for whole group contrasts of interest.

Cluster size	Peak MNI	Peak *t*	Anatomical description
	*x*, *y*, *z*		
Arithmetic [large + small] > Plus1			
457	−19, −100, −1	6.55	L middle occipital lobe
1170	−32, −6, 47	6.11	L MFG and precentral gyrus
242	20, −95, 2	5.59	R occipital lobe, calcarine sulcus
168	28, 29, −1	5.51	R IFG and insula
619	−49, −45, 47	5.21	L IPL
161	−29, 26, 2	5.17	L insula
69	33, −1, 55	4.57	R MFG
188	−37, 4, 27	4.31	L IFG *orbitalis*
122	48, 36, 32	4.23	R MFG
31	20, 12, 62	4.11	R SFG
170	−34, 51, 15	4.06	L MFG
99	1, 19, −11	−4.34	L ACC
Matching [Number > Shape]			
9499	33, −70, 32	13.07	Bilateral IPL, SPL, and MOG
6532	−7, 24, 45	8.74	Bilateral MFG and SFG
239	−29, 24, −1	8.37	L insula
59	20, 44, −16	5.99	R SFG
131	6, −1, 30	5.96	R cingulate gyrus
336	6, −28, −3	5.34	R thalamus and hippocampus
134	10, −11, 5	5.30	R thalamus ventral lateral nucleus
190	43, −13, −3	−6.83	R insula
674	−7, 56, −6	−6.80	L MFG and ACC
139	−17, −8, −20	−6.44	L hippocampus and amygdala
339	−24, −50, 10	−6.23	L temporal lobe
269	60, −28, 27	−5.92	R IPL and SMG
286	23, −45, 15	−5.37	R temporal lobe
182	−44, −60, 22	−5.30	L MTG and AG
44	−59, −3, −18	−5.28	L MTG
64	−7, −68, 22	−5.01	L precuneus
44	−39, 31, −13	−5.00	L IFG
47	−42, −13, 2	−4.94	L insula
35	−29, −43, 60	−4.54	L IPL and postcentral gyrus
39	−29, −33, −18	−4.41	L parahippocampal gyrus
77	−9, −45, 35	−4.40	L precuneus and cingulate gyrus
VSWM [VSWM > Control]			
2399	38, −23, 55	11.78	R SPL and postcentral Gyrus
355	30, 26, −3	7.83	R IFG and insula
264	−32, 26, −1	7.27	L IFG and insula
255	−32, −6, 55	6.82	L MFG and precentral gyrus
947	−17, −68, 52	6.62	L SPL and precuneus
276	15, −23, 5	6.56	R thalamus
140	−17, −53, −23	6.47	L cerebellum
429	−9, 12, 55	6.41	L SFG and SMA
141	−46, 4, 30	5.23	L IFG and precentral gyrus
51	30, −8, −1	4.91	R Putamen
92	43, 4, 37	4.63	R MFG, IFG, and precentral gyrus
104	40, 31, 22	4.59	R MFG
42	48, −18, 22	3.91	R insula
29	−29, −48, −8	3.80	L parahippocampal gyrus
2089	−39, −23, 52	−12.37	L precentral and postcentral gyrus
205	18, −55, −21	−7.02	R cerebellum
360	−22, 31, 52	−5.55	L SFG
47	−27, −1, −13	−5.28	L parahippocampal gyrus
247	−7, 66, 10	−5.26	L MFG and SFG
98	−37, 41, −16	−5.20	L MFG and IFG
184	−51, −23, 20	−4.48	L postcentral gyrus
173	−46, −70, 42	−4.34	L angular gyrus
67	−17, −8, −23	−4.26	L parahippocampal gyrus
42	18, 59, 27	−4.05	R SFG

Abbreviations: ACC, anterior cingulate cortex; IFG, inferior frontal gyrus; IPL, inferior parietal lobule; MFG, middle frontal gyrus; SFG, superior frontal gyrus.

For the arithmetic task, the contrast of interest (Small + Large > Plus1) elicited activation in the bilateral occipital, frontal, and insular areas, as well as in the left inferior and superior parietal lobule. The matching task contrast of interest (Number > Shape) recruited a widespread, bilateral network with large clusters of activation in the occipital, parietal and frontal lobes. Finally, the VSWM contrast of interest (VSWM [collapsed over load] > control [collapsed over load]), activated predominantly bilateral frontal and parietal regions. The clear discrepancy in activation between the left and right motor cortex (i.e., positive *t*‐values in the right hemisphere, negative *t*‐values in the left hemisphere; see slice −25 in Figure 3) can be explained by the fact that participants were instructed to press a button with either their left or right thumb for the VSWM conditions, whereas participants always pressed a button with their right thumb for the Control conditions. This resulted in more right button presses for the control condition, more activation in the left primary motor cortex, and negative *t*‐values in the contrast.

#### 3.2.2 Group comparison of main contrasts of interest

**FIGURE 4:**
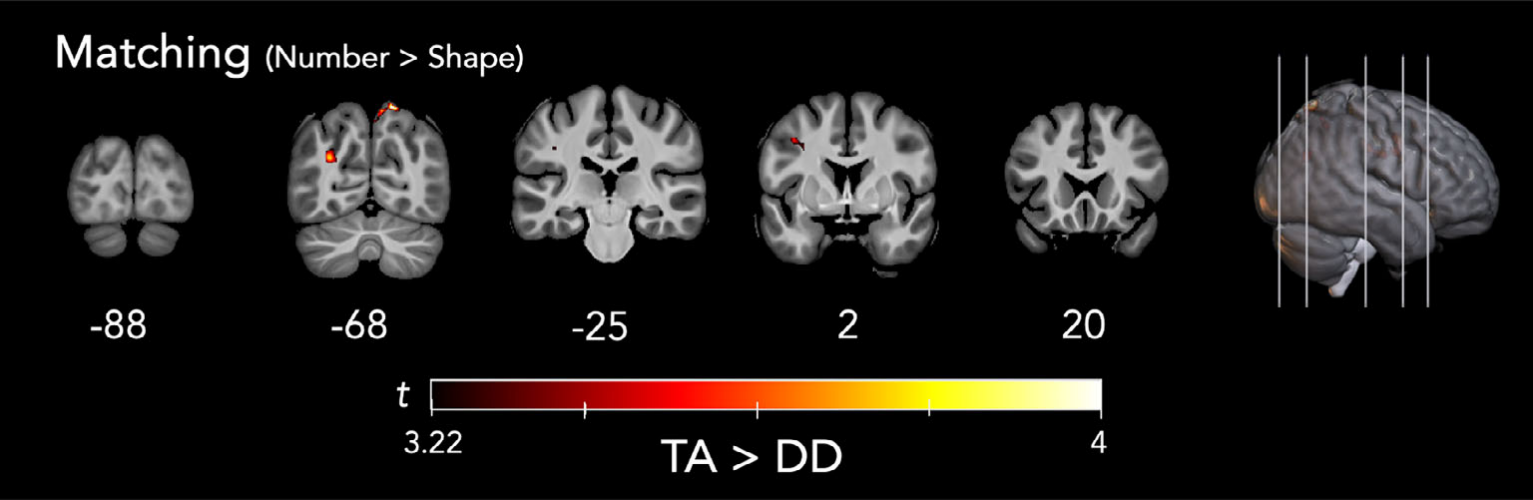
Maps of activation for between‐subjects *t*‐tests (DD > TA) for the matching task, main contrasts of interest (Number > Shape). Significance threshold was *t* = 3.22 at *p* < .001 uncorrected, cluster‐corrected at *k* = 29.

Between‐group analyses comparing TA and DD children did not yield any significant differences for the arithmetic or VSWM tasks. However, there were three brain regions in which TA children showed higher levels of activation compared to DD children in the matching task (Number > Shape): the right superior parietal lobule (rSPL), left precentral gyrus, and left middle occipital gyrus (lMOG; Figure 4; see Table S2 for further cluster details). There were no brain regions where DD children showed higher levels of activation compared to TA children for the matching task.

As a post‐hoc, non‐pre‐registered extension of the analysis of the arithmetic task, we also investigated another variation of the problem size effect, (large > small problems) as well as each problem size versus the implicit baseline (large > fixation; small > fixation). None of these contrasts yielded any significant group differences.

To further explore possible differences in the matching task, we also ran a post‐hoc, non‐preregistered analysis of the simple contrasts of the number condition versus baseline and compared this contrast between groups. This group comparison resulted in four clusters that were more active for the TA group that the DD group including the left precentral gyrus, the left and right middle frontal gyrus, and the left inferior parietal lobe. A table with information on cluster sizes, *t‐*values, and peak coordinates for each task can be found in Table S1. Further, Figure S1 overlays both the Number > Shape (pre‐registered contrast) and Number > Fixation on the same image. It should be noted that the number > fixation contrasts yielded significant voxels in the same R SPL region as the Number > Shape contrast, but that these voxels did not reach the cluster correction threshold of *k* = 29, suggesting that there was some continuity of the difference in parietal activity between‐groups (TA > DD).

#### 3.2.3 Group comparison of number versus face (difficulty‐matched contrast)

Number matching was found to be more difficult for children compared to shape matching (Table 2). To compare number matching to a difficulty‐matched, non‐numerical condition, face matching was included in the experimental paradigm as a third condition. Behavioral results showed that there was no within‐subject difference in performance between number and face matching for either the TA [accuracy *t*(36) = −0.767, *p* = 0.448, Cohen's *d* = −0.126; RT *t*(36) = −1.907, *p* = 0.065, Cohen's *d* = −0.313] or DD group [accuracy *t*(29) = 1.41, *p =* 0.169, Cohen's *d* = 0.257; RT *t*(29) = −1.20, *p* = 0.239, Cohen's *d* = −0.219] (for means by condition, see Table 2). Next, to determine if the difference in task difficulty between number and shape matching drove the obtained group differences in activation for the Number > Shape contrast, we investigated whether there were any group differences in neural activation in the difficulty‐matched Number > Face contrast. The whole‐brain, independent samples *t‐*test showed no brain areas in which TA and DD children differed significantly in activation levels for the Number > Face contrast. To further explore which of the three matching conditions drove results within the three regions demonstrating group differences for the Number > Shape contrast (i.e., the lMOG, lPrecentral Gyrus, and rSPL), we extracted the beta weight associated with each condition versus baseline from each region (Figure S2). In each region, the between‐group difference was greatest for number‐matching with TA children showing greater responses than DD children. Face‐matching also elicited a greater response than shape‐matching in each region, but to a lesser degree. Group differences in the shape‐matching condition were negligible.

### 3.3 Post‐hoc Bayesian approach for group comparison

Given that the pre‐registered analyses comparing DD and TA groups in a frequentist framework were largely null, we next conducted a post‐hoc Bayesian analysis to explore the relative amount of evidence for H_0_ (no group difference) compared to H_1_ (group difference). This Bayesian approach is similar to the voxel‐wise, independent‐sample *t*‐test used in the frequentist approach, but instead yields Bayes factors (BFs) for each voxel. This analysis provides two advantages. First, BFs can be validly interpreted to support a null hypothesis rather than simply fail to reject it. In other words, Bayesian analyses permit us to ask how strong the evidence is that DD and TA groups have similar neural responses to task contrasts of interest. The second advantage is that we can explore results along a continuum of BFs, giving a more nuanced description of how strong evidence is for or against group differences across the whole brain.

To conduct this analysis, we followed Han and Park's guide for second‐level Bayesian inference (Han & Park, 2018) to derive posterior probability maps in SPM12 (Friston & Penny, 2003). The Bayesian second‐level analyses were conducted on the same preprocessed data and first‐level analyses reported above. As Bayesian independent‐samples group comparisons are limited to one‐sided tests, we conducted two analyses for each task contrast of interest, TA > DD and DD > TA, and present the combined results. Results were manually translated from SPM's default of log(BF) to raw BF to more easily compare results to standard BF interpretations. Interpretation heuristics were based on Jeffreys' (1998) original suggestion with one additional distinction. BFs between 3 and 10, which Jeffreys interpreted as moderate evidence, were broken down into the categories of 3–6 “moderate” and 6–10 “substantial” to provide another level of granularity. Posterior probability maps were created with the default effect size threshold (Cohen's *d* = 1.0), no BF threshold, and no voxel extent threshold to visualize complete maps. Raw posterior probability maps have been archived on neurovault **(** https://identifiers.org/neurovault.collection:10338).

#### 3.3.1 Bayesian group comparison of main contrasts of interest

**FIGURE 5:**
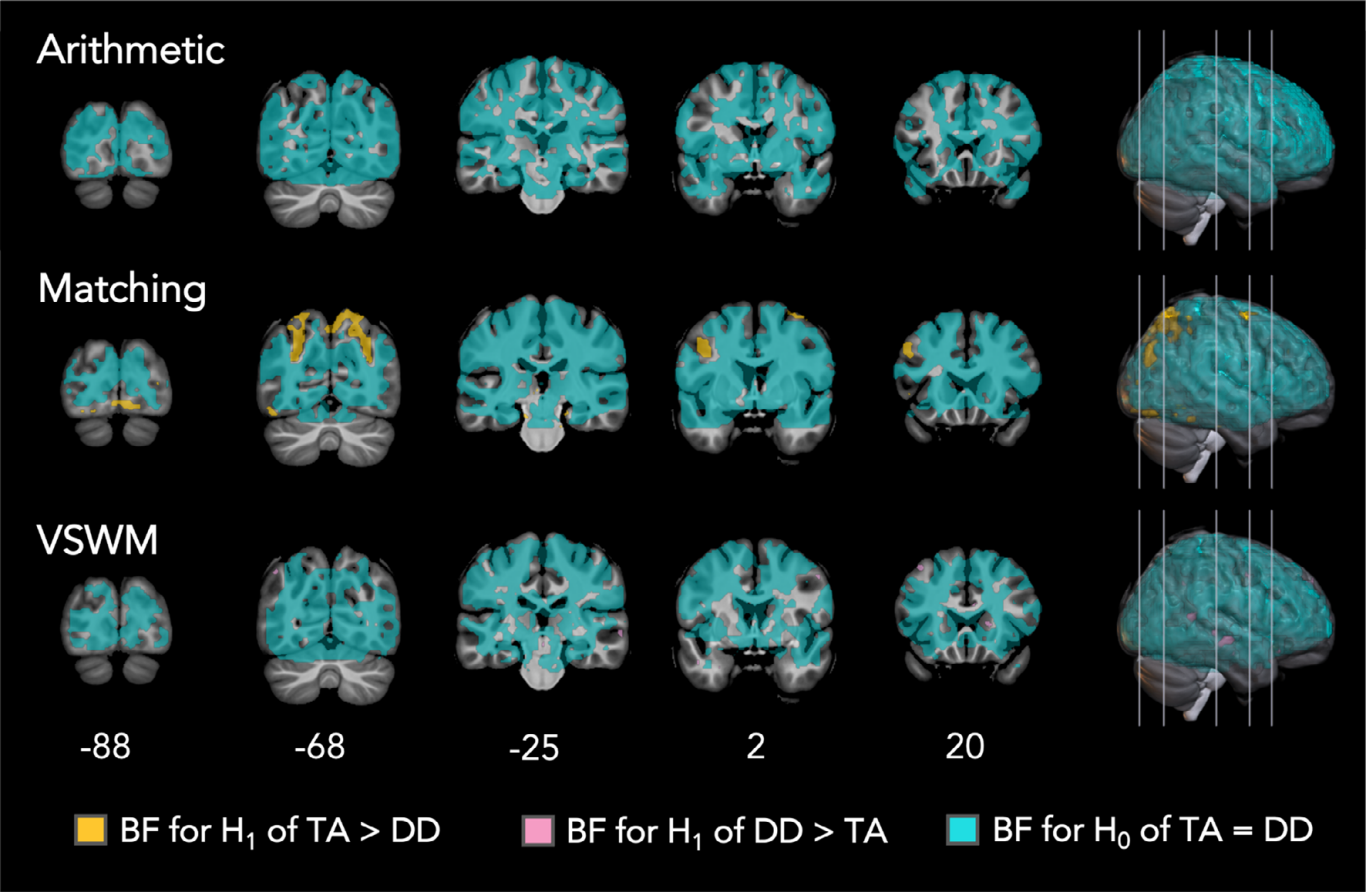
Bayesian achievement group contrasts for all three fMRI task contrasts of interest showing voxels indicating moderate and above evidence (BFs >3) for the hypothesis that typically achieving (TA) children have greater task‐related activity than children with developmental dyscalculia (DD) [orange], moderate and above evidence for the hypothesis that children with DD have greater task‐related activity than TA children [pink], and moderate and above evidence (BF <1/3) for achievement groups having the same task‐related activity [teal]. Note, voxels were only colored as teal if the value was below 1/3 for both the TA > DD and DD > TA comparisons. Neurological convention (right is right).

As in the frequentist approach, the first‐level contrasts of interest for the independent‐sample *t*‐test comparing children with DD to their TA peers were as follows: Arithmetic (Large + Small > Plus1), Matching (Number > Shape), and VSWM (task (collapsed over load) > control (collapsed over load)). Results from all tasks are presented in Figure 5 and Table 4. In Figure 5, BFs have been labeled with separate colors that indicate three categories: (1) greater activation for TA children (2) greater activation for DD children, and (3) TA children and DD children did not differ. Because two independent‐sample *t*‐tests were run, a voxel was only considered as having evidence for H_0_ if the BF was <1/3 in both maps. BF values of 3 (H_1_ is three times more likely than H_0_) and 1/3 (H_1_ is three times less likely than H_0_) correspond to a “moderate” or above level of evidence for an effect. Voxels with BFs between 1/3 and 3 were not given a color as BFs within this range indicate only anecdotal evidence. Table 4 presents a more fine‐grained breakdown of BFs within each color. With this method, we hoped to observe the strength of evidence across the whole brain that actually supported the null findings reflected in the pre‐registered frequentist approach.

**TABLE 4:** Bayes factor bins for task‐based achievement group comparison results.

				Arithmetic	Matching	VSWM
	BF+0 (TA > DD)	Interpretation		% of voxels	% of voxels	% of voxels
For H_1_	>20	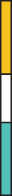	Very strong	0	<0.1	0
	10–20		Strong	0	<0.1	0
	6–10		Substantial	0	0.2	0
	3–6		Moderate	0	0.4	<0.1
	1–3		Anecdotal	0.5	1.3	0.2
For H_0_	1/3–1		Anecdotal	18.8	15.9	3.5
	1/6–1/3		Moderate	38.5	14.8	9.6
	1/10–1/6		Substantial	24.2	10.6	11.6
	1/20–1/10		Strong	13.7	12.8	18.2
	<1/20		Very strong	4.3	38.0	57.0
			Total	100	100	100

	BF0+ (TA < DD)	Interpretation		% of voxels	% of voxels	% of voxels
For H_1_	>20	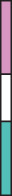	Very Strong	0	0	0
	10–20		Strong	0	0	<0.1
	6–10		Substantial	0	0	<0.1
	3–6		Moderate	<0.1	0	0.3
	1–3		Anecdotal	<0.1	<0.1	4.7
For H_0_	1/3–1		Anecdotal	9.2	0.3	23.5
	1/6–1/3		Moderate	33.4	0.9	23.0
	1/10–1/6		Substantial	25.5	1.4	15.1
	1/20–1/10		Strong	21.4	3.2	15.1
	<1/20		Very strong	10.3	94.1	18.3
			Total	100	100	100

*Note*: Color bars correspond to voxel in Figure 5. All Bayesian contrasts were one‐tailed as noted by BF+0. Top panel, TA > DD. Bottom panel, DD > TA.

Abbreviations: BF, Bayes factor; VSWM, visuo‐spatial working memory.

##### Arithmetic

For the arithmetic task, a total of 95,637 voxels were included in the analysis across all participants. Of these, only three voxels had a BF >3, where the DD group showed greater task‐related activity than the TA group, or <0.001% of the whole brain (Figure 5, top). In the one‐tailed test of TA > DD, 80.7% of voxels had moderate or stronger support for H_0_. In the one‐tailed test of DD > TA, 90.6% of voxels had moderate or stronger support for H_0_. Only 19.3% of voxels showed anecdotal support for H_1_ or H_0_ collectively. Overall, this result provides support for equivalency of arithmetic‐related activity between TA and DD groups.

##### Matching

For the matching task, a total of 90,425 voxels were included. Of these, about 2% had a BF >3 for the TA > DD contrast, or 1792 voxels, demonstrating greater activity for the TA group in the Number > Shape contrast than the DD group (Figure 5, middle). Of these, 152 voxels were categorized as strong (BF 10–20) and 50 were categorized as very strong (BF > 20). These voxels were primarily located in the bilateral IPS (spanning both the superior and inferior sides of the IPS from the posterior portion of the sulcus adjacent to the occipital lobe all the way to the postcentral gyrus), the left ventrolateral prefrontal cortex (including multiple subregions of the inferior frontal gyrus), and the bilateral inferior occipital lobes extending from the lingual gyrus to the anterior inferior temporal gyrus. The highest BFs were located in the middle of the IPS bordering the superior parietal lobule. No voxels were categorized as having moderate or stronger support for DD > TA. In fact, 99.6% of voxels showed moderate or stronger support for H_0_ in this direction. Overall, this result was consistent with the frequentist results, showing three principal clusters where there was strong or very strong evidence that TA children had greater Number > Shape activity during the matching task.

##### Visuo‐spatial working memory

For the VSWM task (Figure 5, bottom, Table 4), a total of 89,582 voxels were included in the analysis across all participants. For the TA > DD contrast, only two voxels had a BF >3, 3.7% showed anecdotal evidence for either H_0_ or H_1_, and 96.4% had BF <1/3 for H_0_. For the DD > TA contrast, 329 voxels (0.37%) had a BF >3, most being within the moderate support range (0.3% within BF 3–6). The most prominent among these regions was a bilateral cluster of voxels (peak MNI coordinates *R* = 64, −16, −8, *L* = −62, −22, −8) in the middle temporal gyrus (MTG), which each had voxels in the substantial and strong BF range (only 39 voxel total across both clusters, 34 substantial, and 5 strong), but no voxels in the very strong range. Overall, given that these results in the MTG were not very strong and would not exceed the *k* = 29 cluster‐threshold implemented in the frequentist results, the case for a meaningful group difference between DD and TA children in the VSWM task is not very strong. Still, only 71.5% of voxels in the DD > TA contrast provided moderate or stronger evidence for H_0_, so the case for a whole‐brain null result is also not very strong.

#### 3.3.2 Bayesian group comparison of follow‐up contrast of interest (matching, Number > Face)

**FIGURE 6:**
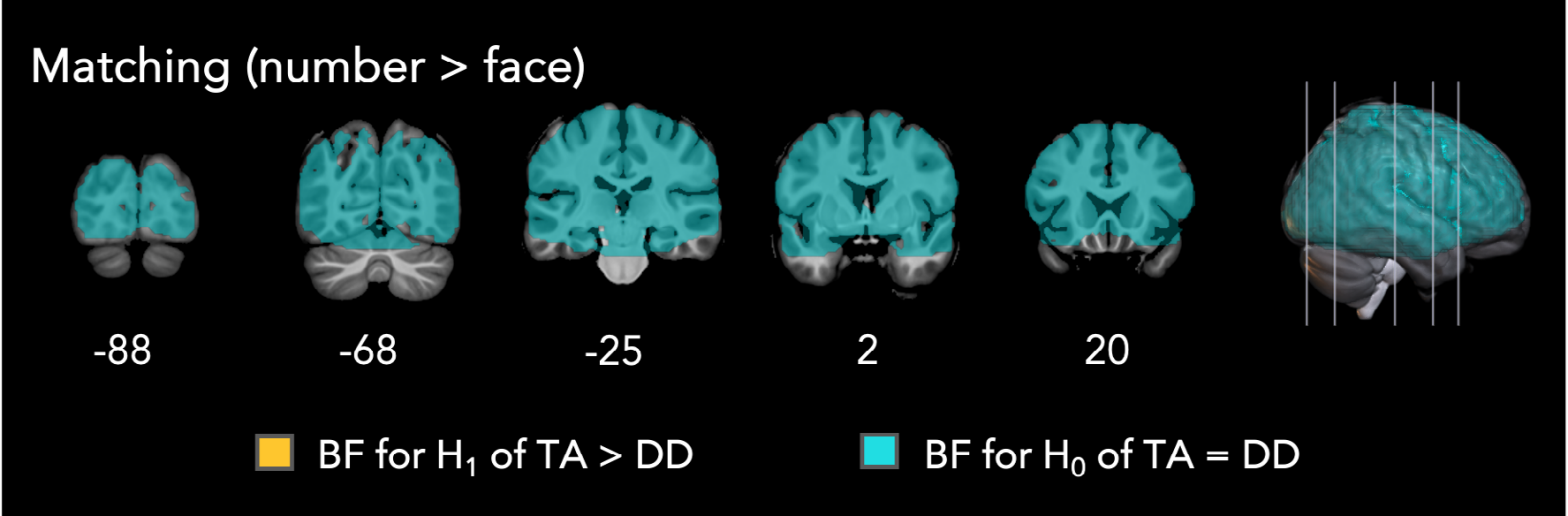
Bayesian achievement group contrasts for difficulty matched follow‐up contrast of interest in the matching task (Number > Face). Colored voxels indicate moderate and above evidence (BFs above 3) for the hypothesis that typically achieving (TA) children have greater task‐related activity than children with developmental dyscalculia (DD) [orange] and moderate and above evidence (BF <1/3) for achievement groups having the same task‐related activity [teal]. Note, voxels were only colored as teal if the value was below 1/3 for both the TA > DD and DD > TA comparisons. Neurological convention (right is right).

With this difficulty‐matched contrast, results largely supported H_0_. In the TA > DD direction, 97.3% of voxels provided moderate or stronger evidence in support of H_0_ (Figure 6, Table 5). Only one voxel was categorized as having moderate support for a group difference in this direction and the remaining voxels were anecdotal in their support. In the DD > TA direction, 98.2% of voxels provided moderate or stronger evidence in support of H_0_. All remaining voxels showed anecdotal evidence. Taken together, this supports the hypothesis that there were no group differences in the Number > Face condition of the matching task. Consequently, in line with the frequentist results, group differences were only observed when the control condition was less difficult than the number matching condition.

**TABLE 5:** Bayes factor bins for achievement group comparison for the matching task contrasting numbers > faces.

				Matching
	BF_+0_ (TA > DD)	Interpretation		% of voxels
For H_1_	>20	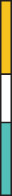	Very strong	0
	10–20		Strong	0
	6–10		Substantial	0
	3–6		Moderate	<0.1
	1–3		Anecdotal	<0.1
For H_0_	1/3–1		Anecdotal	2.4
	1/6–1/3		Moderate	4.0
	1/10–1/6		Substantial	4.2
	1/20–1/10		Strong	7.3
	<1/20		Very Strong	81.8
			Total	100

	BF0+ (TA < DD)	Interpretation		% of voxels
For H_1_	>20	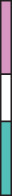	Very Strong	0
	10–20		Strong	0
	6–10		Substantial	0
	3–6		Moderate	0
	1–3		Anecdotal	<0.1
For H_0_	1/3–1		Anecdotal	1.7
	1/6–1/3		Moderate	3.5
	1/10–1/6		Substantial	3.8
	1/20–1/10		Strong	6.7
	<1/20		Very strong	84.2
			Total	100

*Note*: Top panel = TA > DD. Bottom panel = DD > TA. Color bars correspond to voxel in Figure 5. All Bayesian contrasts were one‐tailed as noted by BF+0.

Abbreviation: BF, Bayes factor.

## 4 DISCUSSION

The current, pre‐registered study investigated differences in brain activation elicited by number processing, arithmetic, and VSWM tasks between typically achieving children (TA) and children with developmental dyscalculia (DD). Previous studies have lacked consensus, often reporting both increased and decreased activation for DD children compared to their TA peers across these tasks, with some recent coherence of decreased activity for DD in the right anterior IPS, presented through meta‐analyses (Martinez‐Lincoln et al., 2023; Tablante et al., 2023). One reason for this inconsistency may be that previous studies were challenged by limitations in sample size and inconsistent inclusion criteria for DD. Furthermore, no study had simultaneously investigated all three tasks in the same sample to compare key mechanisms associated with math skill development.

Whole‐group analyses showed that all three tasks elicited neural activity throughout a broad network of fronto–parietal brain areas. These findings converge with recent reviews and meta‐analyses on the neural networks involved in number processing, arithmetic, and VSWM (Arsalidou et al., 2018; Hawes et al., 2019; Klingberg, 2006; Peters & De Smedt, 2018), supporting the validity of the current study's experimental paradigms.

Between‐group analyses on the other hand, showed no differences in neural activation between TA children and children with DD for the arithmetic and the VSWM tasks. These findings contrast with previous literature showing either decreased or increased neural activation for DD children for arithmetic (Peters et al., 2018; Rosenberg‐Lee et al., 2015), and decreased neural activation for DD children for VSWM (Rotzer et al., 2009). It is unlikely that these null findings are the result of a lack of statistical power for multiple reasons. First, given the fact that the current sample size is larger than any previous study, we should in fact have increased power to pick up on subtle group differences. Second, the Bayesian analyses show that there is enough power to suggest mostly strong evidence for the null (i.e., group similarity).

In contrast to the null findings in the arithmetic and VSWM tasks, the number processing task did elicit group differences. There were three regions in the matching task where TA children had a greater difference in activation between number and shape than did DD children, including the right superior parietal lobule (rSPL), left precentral gyrus, and the lMOG. While the left precentral gyrus did not show strong BFs in the Bayesian analysis, values in the lMOG ranged from 20 to 46, and peak values in the rSPL were over 400, indicating very strong evidence of a group difference. After extracting beta‐weights from each of the three clusters to compare activation levels within each condition, the main group‐level difference appeared to derive from number processing. Mean activation levels were very similar across groups for the shape condition, slightly greater for TA than DD for the face condition, and much greater for TA than DD in the number condition. Quantitatively, these values explain why the number versus shape condition elicited group differences while the number versus face condition did not. Considered together with the results from the Bayesian analysis, there appears to be a robust group difference in brain activation of the superior parietal lobe that is greatest during number processing but is somewhat attenuated during an equally difficult, but non‐numerical comparison task.

This finding of decreased superior parietal activation for DD children in the Number > Shape and Number > Fixation contrasts is convergent with a number of studies that show lower activation of superior parietal structures in DD relative to TA during number processing including the superior parietal lobule and precuneus (Kaufmann et al., 2011) and the IPS (Price et al., 2007), most consistently right‐lateralized. These findings have typically been interpreted to support the magnitude processing deficit account of DD (Iuculano et al., 2008). However, these same parietal structures have also been associated with more domain‐general functions, such as attentional control (Connolly et al., 2016; for a review of this critique, see Wilkey & Ansari, 2020). To control for group differences in domain‐general function, the current study set up a stringent, a priori, difficulty‐matched control condition (i.e., face processing). Results of the difficulty‐matched Number > Face contrast showed no significant group differences in the frequentist approach and only anecdotal evidence in the Bayesian results (BFs ≈ 2) in favor of a TA > DD group difference. Altogether, the current results do not support a magnitude‐specific processing deficit account of DD, which is in line with a growing number of behavioral studies (Astle & Fletcher‐Watson, 2020; Mammarella et al., 2021).

The current study results also do not lend support to an account of DD being associated with neural mechanisms of VSWM. While there is substantial behavioral evidence that children with math learning difficulties perform more poorly on VSWM tasks (Geary, 1993; Szucs et al., 2013), and some evidence that task‐related recruitment of neural resources differs (McCaskey et al., 2017; Rotzer et al., 2009), the current study does not show such a group difference. Here, the TA and DD groups do not differ significantly in the VSWM dot‐matrix task behaviorally or in associated neural activity. Still, it has been suggested that deficits in working memory of various types, including VSWM, may lead to math learning difficulties in only a subset of individuals with DD (Siegel & Ryan, 1989; Skagerlund & Träff, 2016; Szűcs, 2016). If this is the case, the coarse group‐difference split in the current study could have washed out an effect present in only a subtype of DD. However, the largest behavioral characterization of DD to date also failed to detect a difference in VSWM (Mammarella et al., 2021).

More broadly, the idea that DD is heterogeneous in nature, where one individual with math difficulties is very different in cognitive profile from the next has substantial support in the literature (Kucian & von Aster, 2015; Siemann & Petermann, 2018; Szűcs, 2016). For example, some research points to a distinction between individuals who have an impairment working with nonsymbolic numerical magnitudes (representing an impaired approximate number system) while others may have difficulty with symbolic number (represented impaired access to number through symbolic representation, also known as the access deficit hypothesis) (Skagerlund & Träff, 2016). Other research has pointed to even more fine‐grained subgroups characterized by a mixture of domain‐specific processing issues (e.g., approximate number sense, subitizing, enumeration, number comparison, and mental number line representation) and domain‐general processing issues (e.g., VSWM and verbal working memory; Chan & Wong, 2020). Unfortunately, the current study's sample size does not allow for an analysis of possible subtypes of DD that may mask real differences between the neural response profiles of the DD and TA groups when analyzed with mean differences between two groups. Further, we cannot rule out the possibility that some individuals with DD in the current sample also had comorbid developmental disabilities not captured by the current testing battery, such as difficulty reading or a more generalized intellectual impairment that led to increased heterogeneity in the sample.

One possibility for the current study's failure to support either the magnitude deficit account or VSWM account of DD is methodological. It may be that a univariate contrast between groups is not sensitive enough to capture differences in the neural activity associated with each type of processing. It is possible that a more sensitive analysis, or one that captures a different aspect of the neural activity, such as patterns in multiple voxels or functional networks or is limited to symbolic or non‐symbolic number, could yield significant results. We characterized robust behavioral differences in achievement measures and fMRI task behaviors between children with DD and their TA counterparts that must have origins in the neurocognitive mechanisms used in the associated tasks. Ultimately, it stands to reason that there are differences in neural processing of mathematical information that must account for these behavioral differences that simply were not captured reliably with a traditional approach. Differences in behavior necessarily correspond to differences in brain activity. The current results simply indicate that using a common fMRI technique, we did not reliably characterize differences in neural signature between DD and TA groups that correspond to the theoretical frameworks for characterizing DD that we tested. Still, this potential for future findings does not explain the current study's divergence from univariate fMRI results in the published literature. Overall, the main contribution detailed in the current results is that the most commonly used analytic approach conducted in the largest DD sample to date did not yield results consistent with existing narratives in the neuroimaging literature on math learning difficulties.

The current study makes two further contributions to the field. First, the level of detail provided by our Bayesian approach allowed us to evaluate support for group similarity as well as group differences, and to further describe the robustness of those results in terms of probability. In the process, we were able to more clearly delineate null results that are inconclusive due to a lack of power or high sample variability from those null results that strongly indicate a similarity between groups in brain activation in a given brain region. Second, the current dataset has been archived publicly for secondary data analysis, which we hope will increase opportunities for replicability and further analysis of the same sample. We believe that pre‐registered, transparent analysis, full reporting of results, and open data practices are key for building a consensus around the neurocognitive correlates of DD.

Overall, these results suggest that the most common methods used to uncover the neural correlates of math learning difficulties, namely univariate group contrasts of brain function in behaviorally relevant tasks that correspond to dominant theories of DD, may be insufficient. It is possible that the neural markers of learning difficulties are not as specific as originally thought. With a well‐characterized sample of the largest size to date comparing TA and DD children, and three common fMRI tasks, the current study failed to support most previous accounts for the neural basis of DD. Given these findings, we suggest the field proceeds on two fronts. First, it is likely that fully understanding math learning difficulties will require multivariate and network‐based approaches that are capable of capturing differences in neurocognitive mechanisms beyond what we would expect in core deficit accounts of DD. Second, replicability of findings will depend on the adoption of larger, more well‐defined samples, stricter thresholds for positive findings, and transparency in analytic approach. Together, we believe that these improvements to the research framework can lead to a deeper understanding of the heterogeneous, persistent sources of math learning difficulties.

## FUNDING INFORMATION

The study was funded by the National Research Foundation (Singapore), NRF2016‐SOL001‐003.

## CONFLICT OF INTEREST STATEMENT

The authors have declared that no competing interests exist.

## Supporting information


**Figure S1.** Maps of activation for between‐subjects *t*‐tests (TA > DD) for the matching task, main contrasts of interest (number > shape, blue) and exploratory contrast (number > fixation, orange). Significance threshold was *t* = 3.22 at *p* < .001 uncorrected, cluster‐corrected at k = 29.

**Figure S2.** Beta‐weights extracted from ROIs showing significant between‐group differences in pre‐registered contrasts of interest (Number > Shape and Number > Face). To extract z‐normalized beta weights, first‐level contrasts of each condition were created (e.g., number vs baseline) and beta‐weights for each condition were estimated for each condition. L MOG = left middle occipital gyrus; L Precentral = left precentral gyrus; R SPL = right superior parietal lobule. DD = developmental dyscalculia, TA = typically achieving.

**Table S1.** Significant Clusters for Whole Group Contrasts of Interest

**Table S2.** Significant Clusters for Between‐Group Contrasts of Interest

**Data S1.** Supporting Information.

## Data Availability

The data that support the findings of this study are openly available in OSF at https://osf.io/vsr8b. The MRI data are openly available. The anonymized, de‐faced fMRI data and supporting behavioural data have been publicly archived on OpenNeuro.org at doi:10.18112/openneuro.ds004791.v1.0.0.
