# Supplementary figures and images for: Developmental dyscalculia is not associated with atypical brain activation: A univariate fMRI study of arithmetic, magnitude processing, and visuospatial working memory
Source: Hum Brain Mapp. 2023 Nov 1;44(18):6308–25. doi: 10.1002/hbm.26495 (PMC10681641; doi:10.1002/hbm.26495)

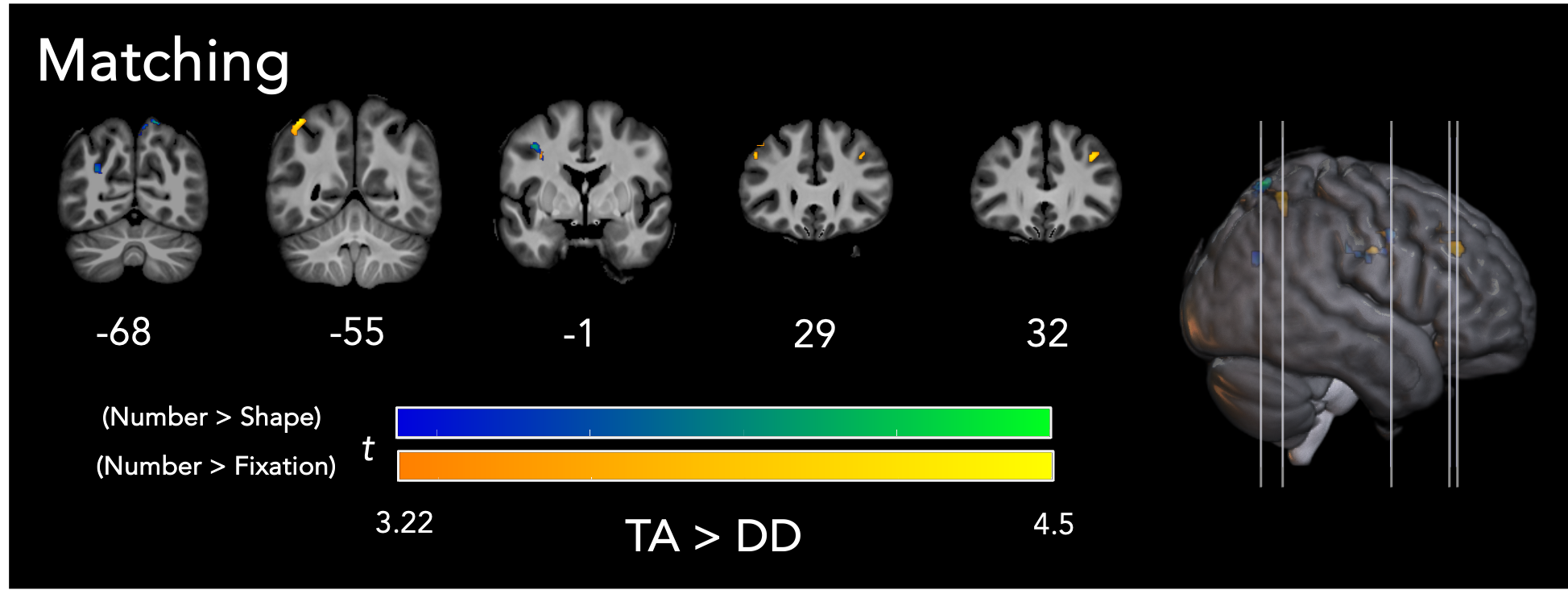

Supplement: Supplementary file 1 — Figure S1. Maps of activation for between‐subjects t‐tests (TA > DD) for the matching task, main contrasts of interest (number > shape, blue) and exploratory contrast (number > fixation, orange). Significance threshold was t = 3.22 at p < .001 uncorrected, cluster‐corrected at k = 29. [file HBM-44-6308-s003.png]

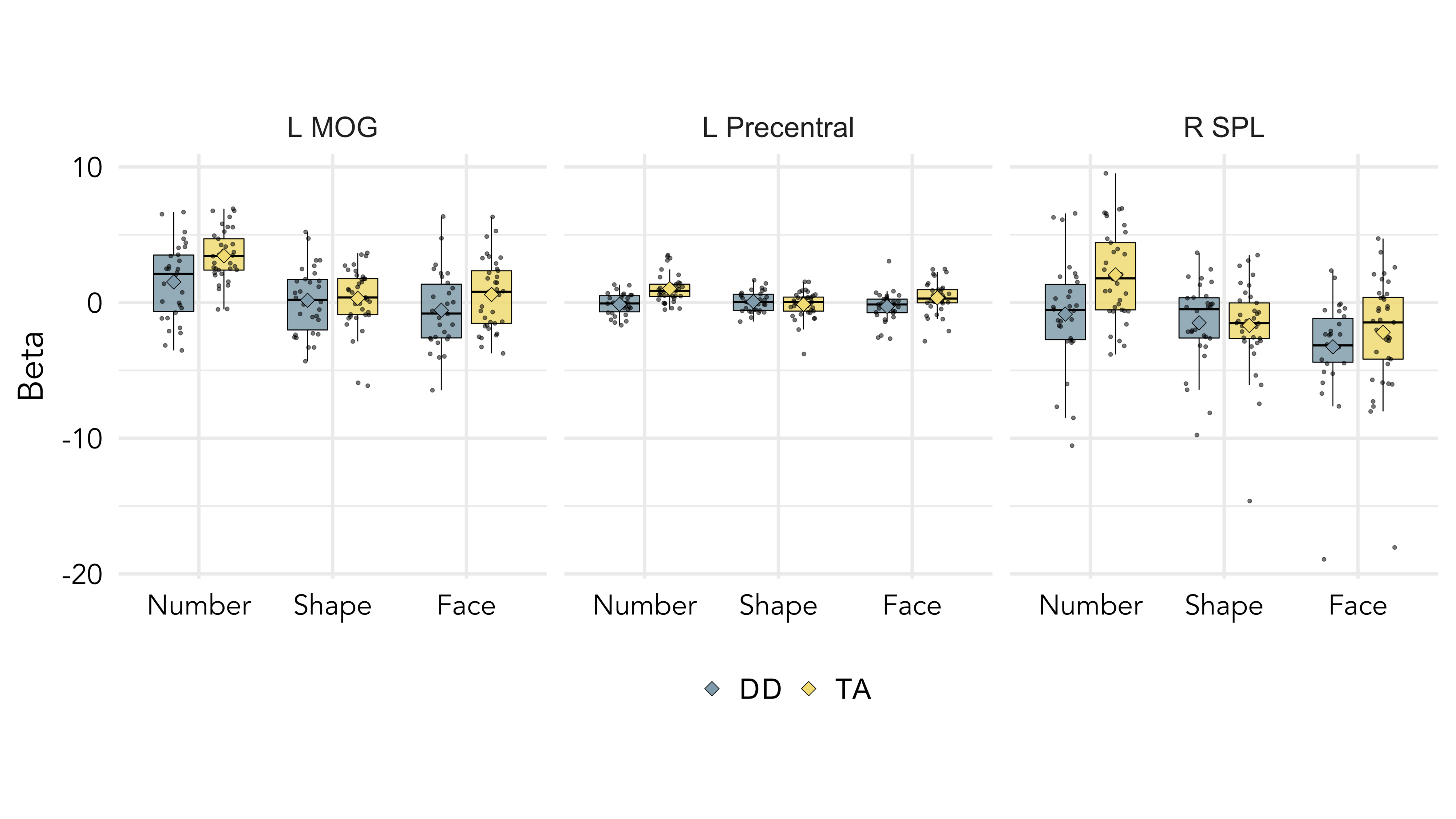

Supplement: Supplementary file 2 — Figure S2. Beta‐weights extracted from ROIs showing significant between‐group differences in pre‐registered contrasts of interest (Number > Shape and Number > Face). To extract z‐normalized beta weights, first‐level contrasts of each condition were created (e.g., number vs baseline) and beta‐weights for each condition were estimated for each condition. L MOG = left middle occipital gyrus; L Precentral = left precentral gyrus; R SPL = right superior parietal lobule. DD = developmental dyscalculia, TA = typically achieving. [file HBM-44-6308-s005.tiff]
