# Supplementary material for: Developmental dyscalculia is not associated with atypical brain activation: A univariate fMRI study of arithmetic, magnitude processing, and visuospatial working memory
Source: Hum Brain Mapp. 2023 Nov 1;44(18):6308–25. doi: 10.1002/hbm.26495 (PMC10681641; doi:10.1002/hbm.26495)
Supplement: Supplementary file 3 — Table S1. Significant Clusters for Whole Group Contrasts of Interest [file HBM-44-6308-s002.docx]

| **Table S1.** Significant Clusters for Whole Group Contrasts of Interest | | | |
| --- | --- | --- | --- |
| **Cluster size** | **MNI**  **x, y, z** | **Peak *t***  *minor maxima* | **Anatomical description** |
|  |  |  |  |
| **Arithmetic** [Large + Small] > Plus1 | | |  |
|  |  |  |  |
| **457** | **-19, -100, -1** | **6.55** | **L middle occipital lobe** |
|  | -12, -82, 4 | 5.02 |  |
|  | -39, -85, -13 | 4.66 |  |
| **1170** | **-32, -6, 47** | **6.11** | **L MFG & precentral gyrus** |
|  | -9, 14, 50 | 5.93 |  |
|  | -9, 29, 32 | 5.92 |  |
| **242** | **20, -95, 2** | **5.59** | **R occipital lobe, calcarine sulcus** |
|  | 35, -87, -8 | 5.12 |  |
| **168** | **28, 29, -1** | **5.51** | **R IFG & insula** |
|  | 33, 22, -6 | 5.06 |  |
| **619** | **-49, -45, 47** | **5.21** | **L IPL** |
|  | -32, -48, 37 | 4.82 |  |
|  | -34, -63, 54 | 4.74 |  |
| **161** | **-29, 26, 2** | **5.17** | **L insula** |
|  | -32, 19, -6 |  |  |
| **69** | **33, -1, 55** | **4.57** | **R MFG** |
| **188** | **-37, 4, 27** | **4.31** | **L IFG *orbitalis*** |
|  | -49, 9, 42 | 4.25 |  |
|  | -51, 9, 24 | 4.23 |  |
| **122** | **48, 36, 32** | **4.23** | **R MFG** |
|  | 38, 29, 30 | 3.92 |  |
| **31** | **20, 12, 62** | **4.11** | **R SFG** |
| **170** | **-34, 51, 15** | **4.06** | **L MFG** |
|  | -34, 56, 7 | 3.99 |  |
|  | -44, 44, 7 | 3.96 |  |
|  |  |  |  |
| **99** | **1, 19, -11** | **-4.34** | **L ACC** |
|  | 3, 4, -10 | 3.91 |  |
|  | -9, 24, -8 | 3.62 |  |
|  |  |  |  |
|  |  |  |  |
| **Matching** [Number > Shape] | | |  |
|  | | |  |
| **9499** | **33, -70, 32** | **13.07** | **Bilateral IPL, SPL & MOG** |
|  | -27, -65, 47 | 11.76 |  |
|  | 25, -92, 2 | 11.62 |  |
| **6532** | **-7, 24, 45** | **8.74** | **Bilateral MFG & SFG** |
|  | -39, 4, 34 | 8.27 |  |
|  | 6, 26, 44 | 8.17 |  |
| **239** | **-29, 24, -1** | **8.37** | **L insula** |
| **59** | **20, 44, -16** | **5.99** | **R SFG** |
|  | 22, 51, -13 | 4.30 |  |
|  | 25, 58, -8 | 3.54 |  |
| **131** | **6, -1, 30** | **5.96** | **R cingulate gyrus** |
|  | -4, 12, 24 | 5.60 |  |
|  | 6, 12, 24 | 4.93 |  |
| **336** | **6, -28, -3** | **5.34** | **R thalamus & hippocampus** |
|  | 22, -29, -3 | 5.03 |  |
|  | 5, -33, -18 | 5.00 |  |
| **134** | **10, -11, 5** | **5.30** | **R thalamus ventral lateral nucleus** |
|  | 10, -23, 12 | 4.23 |  |
|  | 20, -6, 4 | 4.02 |  |
|  |  |  |  |
| **190** | **43, -13, -3** | **-6.83** | **R insula** |
|  | 50, 2, -8 | -5.34 |  |
| **674** | **-7, 56, -6** | **-6.80** | **L MFG & ACC** |
|  | -4, 26, -8 | -6.59 |  |
|  | 6, 49, -13 | -5.51 |  |
| **139** | **-17, -8, -20** | **-6.44** | **L hippocampus & amygdala** |
|  | -24, 7, -16 | -3.81 |  |
| **339** | **-24, -50, 10** | **-6.23** | **L temporal lobe** |
|  | -19, -43, 17 | -5.74 |  |
|  | -14, -35, 20 | -5.31 |  |
| **269** | **60, -28, 27** | **-5.92** | **R IPL & SMG** |
|  | 67, -29, 19 | -4.79 |  |
|  | 47, -36, 27 | -4.56 |  |
| **206** | **-56, -28, 27** | **-5.43** | **L IPL & SMG** |
|  | -66, -33, 24 | -4.99 |  |
| **286** | **23, -45, 15** | **-5.37** | **R temporal lobe** |
|  | 33, -45, -1 | -5.36 |  |
|  | 10, -33, 17 | -4.94 |  |
| **182** | **-44, -60, 22** | **-5.30** | **L MTG & AG** |
| **44** | **-59, -3, -18** | **-5.28** | **L MTG** |
| **64** | **-7, -68, 22** | **-5.01** | **L precuneus** |
|  | -12, -58, 14 | -3.32 |  |
| **44** | **-39, 31, -13** | **-5.00** | **L IFG** |
|  | -30, 33, -8 | -3.85 |  |
| **47** | **-42, -13, 2** | **-4.94** | **L insula** |
|  | -49, -8, 0 | -4.02 |  |
| **35** | **-29, -43, 60** | **-4.54** | **L IPL & postcentral gyrus** |
| **39** | **-29, -33, -18** | **-4.41** | **L parahippocampal gyrus** |
| **77** | **-9, -45, 35** | **-4.40** | **L precuneus & cingulate gyrus** |
|  |  |  |  |
|  |  |  |  |
| **VSWM [**VSWM > Control] | | |  |
|  |  |  |  |
| **2399** | **38, -23, 55** | **11.78** | **R SPL & postcentral Gyrus** |
|  | 15, -68, 52 | 7.17 |  |
|  | 25, -1, 54 | 6.80 |  |
| **355** | **30, 26, -3** | **7.83** | **R IFG & insula** |
| **264** | **-32, 26, -1** | **7.27** | **L IFG & insula** |
|  | -37, 17, -8 | 5.75 |  |
|  | -32, 19, 10 | 5.27 |  |
| **255** | **-32, -6, 55** | **6.82** | **L MFG & precentral gyrus** |
| **947** | **-17, -68, 52** | **6.62** | **L SPL & precuneus** |
|  | -32, -50, 42 | 5.46 |  |
|  | -32, -58, -54 | 5.28 |  |
| **276** | **15, -23, 5** | **6.56** | **R thalamus** |
|  | 10, -21, 12 | 6.03 |  |
|  | -2, -26, -3 | 4.87 |  |
| **140** | **-17, -53, -23** | **6.47** | **L cerebellum** |
|  | -7, -63, -18 | 4.73 |  |
| **429** | **-9, 12, 55** | **6.41** | **L SFG & SMA** |
|  | -9, 17, 47 | 6.09 |  |
|  | 8, 29, 32 | 5.36 |  |
| **141** | **-46, 4, 30** | **5.23** | **L IFG & precentral gyrus** |
|  | -49, 2, 40 | 4.17 |  |
| **51** | **30, -8, -1** | **4.91** | **R Putamen** |
|  | 30, -1, 4 | 3.38 |  |
| **92** | **43, 4, 37** | **4.63** | **R MFG, IFG & precentral gyrus** |
|  | 58, 17, 27 | 3.57 |  |
| **104** | **40, 31, 22** | **4.59** | **R MFG** |
|  | 45, 34, 30 | 3.690 |  |
|  | 35, 36, 30 | 3.59 |  |
| **42** | **48, -18, 22** | **3.91** | **R insula** |
|  | 38, -23, 22 | 3.85 |  |
| **29** | **-29, -48, -8** | **3.80** | **L parahippocampal gyrus** |
|  |  |  |  |
| **2089** | **-39, -23, 52** | **-12.37** | **L pre- & postcentral gyrus** |
|  | -9, -26, 47 | -6.06 |  |
|  | 3, -87, 27 | -4.59 |  |
| **205** | **18, -55, -21** | **-7.02** | **R cerebellum** |
| **360** | **-22, 31, 52** | **-5.55** | **L SFG** |
|  | -17, 59, 32 | -5.21 |  |
|  | 3, 29, -20 | -4.79 |  |
| **47** | **-27, -1, -13** | **-5.28** | **L parahippocampal gyrus** |
|  | -29, -8, -3 | -3.36 |  |
| **247** | **-7, 66, 10** | **-5.26** | **L MFG & SFG** |
|  | -4, 31, -13 | -4.92 |  |
|  | 3, 29, -16 | -4.56 |  |
| **98** | **-37, 41, -16** | **-5.20** | **L MFG & IFG** |
|  | -44, 26, -13 | -4.73 |  |
|  | -34, 29, -20 | -4.35 |  |
| **184** | **-51, -23, 20** | **-4.48** | **L postcentral gyrus** |
|  | -61, -23, 20 | -4.39 |  |
|  | -36, -16, 14 | -4.31 |  |
| **173** | **-46, -70, 42** | **-4.34** | **L angular gyrus** |
|  | -49, -68, 32 | -4.08 |  |
|  | -37, -78, 44 | -3.97 |  |
| **67** | **-17, -8, -23** | **-4.26** | **L parahippocampal gyrus** |
|  | -19, -13, -10 | -4.06 |  |
| **42** | **18, 59, 27** | **-4.05** | **R SFG** |
|  | 15, 51, 40 | -3.71 |  |
|  | 20, 51, 32 | -3.49 |  |
|  |  |  |  |
| MFG = middle frontal gyrus; IFG = inferior frontal gyrus; IPL = inferior parietal lobule; SFG = superior frontal gyrus; ACC = anterior cingulate cortex; LGN = lateral geniculate nucleus of the thalamus; Table reports up to 3 local maxima more than 8.0 mm apart. | | | |
