# Supplementary material for: Developmental dyscalculia is not associated with atypical brain activation: A univariate fMRI study of arithmetic, magnitude processing, and visuospatial working memory
Source: Hum Brain Mapp. 2023 Nov 1;44(18):6308–25. doi: 10.1002/hbm.26495 (PMC10681641; doi:10.1002/hbm.26495)
Supplement: Supplementary file 4 — Table S2. Significant Clusters for Between‐Group Contrasts of Interest [file HBM-44-6308-s004.docx]

| **Table S2.** Significant Clusters for Between-Group Contrasts of Interest | | | | |
| --- | --- | --- | --- | --- |
|  | **Peak MNI  (x, y, z)** | **Cluster size** | **Peak *t*** | **Anatomical description** |
|  |  |  |  |  |
| **Matching: Number > Shape** |  |  |  |  |
|  |  |  |  |  |
| TA > DD | (20, -65, 67) | 62 | 4.98 | R SPL & precuneus |
|  | (-42, -23, 35) | 115 | 4.15 | L precentral gyrus |
|  | (-29, -68, 27) | 34 | 3.81 | L MOG |
|  |  |  |  |  |
| **Matching: Number > Fixation** |  |  |  |  |
|  |  |  |  |  |
| TA > DD | (-39, -10, 32) | 57 | 4.09 | L precentral gyrus |
|  | (35, 31, 34) | 37 | 4.15 | R middle frontal gyrus |
|  | (-46, 29, 34) | 45 | 4.14 | L middle frontal gyrus |
|  | (-42, -55, 62) | 34 | 3.81 | L SPL |
|  |  |  |  |  |
| *Note.* L = left, R = right, SPL = superior parietal lobule, MOG = middle occipital gyrus. | | | | |
