# Supplementary material for: Developmental dyscalculia is not associated with atypical brain activation: A univariate fMRI study of arithmetic, magnitude processing, and visuospatial working memory
Source: Hum Brain Mapp. 2023 Nov 1;44(18):6308–25. doi: 10.1002/hbm.26495 (PMC10681641; doi:10.1002/hbm.26495)
Supplement: Supplementary file 5 — Data S1. Supporting Information. [file HBM-44-6308-s001.docx]

**Appendix A. Recruitment Details / Group Categorization / Stability Supplement**

**A.1. Recruitment Details.**

Schools were first contacted to gain approval. If the school consented to the study, parents of the shortlisted participants were contacted via the school in a two-tiered approach. A consent to contact requesting for contact details of the parents was first sent out to the parents. Parents can choose to leave their details to be contacted about the study or return the form with a non-consent to the study. Thereafter, a consent form was sent out to the parents who expressed interest in the study, as well as given detailed information about the study via a call. Parents either consented to allowing their child to: (a) Take part only in the behavioural portion of the study, or (b) Take part in both the behavioural and fMRI portion of the study. Parents who chose the latter answered a short screener to determine their child's eligibility. The consent forms were distributed and collected via the school or in some cases, emailed directly to the parents. Participants who took part in the behavioural portion only were given a small token of appreciation, while those who participated in the fMRI scan additionally received compensation in the form of cash vouchers. Parent and child participation was voluntary. Written informed consent from the parents and child assent were obtained before data collection.

**A.2. Justification for combining LSM and LA children.**

On entry to Grade 1, all children in Singapore complete a screening assessment to determine their need for learning support in mathematics. All children from the original project sample (Ng 2014, 2020) who were subsequently enrolled into the Learning Support for Mathematics (LSM) program in Grade 1 were invited to participate in the current study. In addition, there were children in the SKIP cohort who showed consistent low achievement (bottom 10% of sample) in mathematics across Kindergarten and Grade 1, but who were not identified as in need of learning support. These children were also invited to participate in the study. Analyses comparing the LSM (n = 22) and consistent low achievement (n = 40) groups indicated similar performance profiles at each wave of data collection (see **Table A-1**), supporting our decision to combine them into one developmental dycalculia group (DD). The groups also showed highly similar performance profiles on all additional assessments at Grade 3 (see **Table A-2**).

**Table A-1.** Mean performance (SD) of LSM and consistent low achievement groups on TEMA

| Grade | LSM | Low achievement | *t* | *p* |
| --- | --- | --- | --- | --- |
| Start K1 | 12.47 (7.71) | 11.62 (5.12) | -.47 | .639 |
| End K1 | 16.29 (5.55) | 16.47 (5.25) | .13 | .900 |
| K2* | 20.05 (9.44) | 20.92 (5.44) | .37 | .866 |
| Grade 1* | 28.75 (7.96) | 30.86 (3.79) | 1.12 | .275 |
| Grade 3 | 58.55 (10.70) | 60.90 (7.66) | 1.01 | .319 |

* indicates value for equal variances not assumed.

**Table A-2.** Mean performance (SD) of LSM and consistent low achievement groups on numerical assessments at Grade 3.

| Task | LSM | Low achievement | *t* | *p* |
| --- | --- | --- | --- | --- |
| Number Sets | 2.59 (.89) | 2.40 (.46) | -1.09 | .279 |
| Problem Solving | 36.45 (4.78) | 37.05 (3.53) | .60 | .578 |
| Numerical Operations* | 26.19 (5.33) | 27.23 (3.08) | .82 | .419 |
| Addition Fluency | 20.82 (7.89) | 19.75 (5.40) | -.63 | .530 |
| Subtraction Fluency* | 14.82 (8.19) | 15.55 (5.30) | .38 | .708 |

* indicates value for equal variances not assumed.

**A.3. LPA to check stability of DD and TA groupings.**

Our pre-registered analyses focused on multi-group comparisons (developmental dyscalculia [DD] vs typically achieving [TA]) and multivariate subject classification analyses. These original groupings were formed on the basis of mathematics performance (TEMA) on entry to Grade 1 in 2017. These children were subsequently re-tested for the current study when they were in Grade 3 (2019). There is a possibility that some children respond very well to formal instruction in mathematics on entry to primary school and show rapid growth in math achievement. To ensure the original groupings still held, we conducted some preliminary analyses prior to the original pre-registered analyses.

Before analyzing the fMRI data, we conducted a latent profile analysis (LPA) based on numeracy and mathematics assessment taken at Grade 3 for all children in the sample who completed behavioural testing. LPA is a data driven approach that aims to categorise individuals into a specified number of groups based on their performance profile, and a group prediction for each individual is determined. Five assessments taken at Grade 3 were included in the LPA – Number Sets d prime, WIAT Problem Solving Raw Score, WIAT Numerical Operations raw Score, WIAT Math Fluency Addition raw score, and WIAT Math Fluency Subtraction Raw Score. We started with a 2-profile model on the expectation of stability in math ability, and hence a clear distinction of an DD from a TA profile. Profile 1 included 56 children who were lower achieving on all assessment compared to Profile 2 (78 children), see Table 3. Entropy (.817) for this model was above the recommended level of .80 (Clark & Muthén, 2009). Subsequent chi-square analysis was conducted to determine the association between LPA profile and original (Grade 1) DD vs TA grouping. This revealed a significant association, ꭓ^2^ (1) = 49.80, *p* < .001. Of the original 72 TA children, 62 were in Profile 2 (high ability). Of the original 62 DD children, 46 were in Profile 1 (low ability). Of the children who completed fMRI and had usable data (n = 68, as reported in the main manuscript), 21 of 30 of the original DD children were identified in the low ability profile at Grade 3, and 31 of 38 of the original TA children were identified in the high ability profile at P3. The movement of some children between ability grouping suggested the possibility of 3 profiles (low, average, and high). However, the Lo-Mendell-Rubin adjusted LRT test comparing 2 versus 3-profile solutions suggested no significant improvement in model fit (*p* = .24), and so the 2-profile solution was retained as the preferred model. Given these findings, we decided to maintain the planned group distinctions for the following reasons: (1) this allows for a direct alignment with our preregistered analysis approach and no strong justification emerged for diverging from this plan, (2) a 3-profile solution did not improve model fit over the 2-profile fit, and (3) these groupings retain all children in the analysis, providing increased variability and greater power.

**Table A-3.** Standardised scores on each assessment for each profile group.

| Task | Profile 1 (N = 56). Low performing | Profile 2 (N = 78). High performing |
| --- | --- | --- |
| Number Sets | -.725 | .479 |
| Problem Solving | -.853 | .564 |
| Numerical Operations | -.700 | .469 |
| Addition Fluency | -.779 | .515 |
| Subtraction Fluency | -.885 | .585 |

**Appendix B. Test of Early Mathematics Ability (TEMA-3) details**

This task is useful for identifying children who are behind or ahead of their peers in mathematical thinking, identifying specific strengths and weaknesses, suggesting instructional practices for individual children and documenting mathematical progress (Ginsburg & Baroody, 2003, as cited in Hoffman & Grialou, 2005). It measures children’s informal (acquired outside the context of schooling) and formal mathematics knowledge (skills and concepts learned in school). Informal knowledge is measured through four categories of items: numbering (e.g., verbal counting by ones), number comparisons (e.g., choosing the larger number), calculation (e.g., addition of concrete objects), and concepts (e.g., number constancy). Formal knowledge is also assessed via four categories: numeral literacy (e.g., reading or writing numerals), number facts (e.g., subtraction facts), calculation (e.g., written addition accuracy), and concepts (e.g., written representation of sets). The task has two parallel forms, but only Form A (72 items) was administered in this study. Although this task is suited for children between ages 3 to 8.9, it is also used for identifying the basis of poor mathematical performance in older children. Test administration began at Item A43 (the highest entry point for older children). Each item was either scored as “1” (if the scoring criteria were met) or “0” (if the scoring criteria were not met). Administration was terminated when ceiling (i.e., 5 consecutive scores of zero) and basal (i.e., 5 consecutive scores of “1”) were established. Following the TEMA-3 manual, non-administered items before basal were scored “1” and non-administered items after ceiling were scored ”0”. The dependent measure was the sum of scores on all items below the ceiling. A high score reflects better numeracy skills.

**Appendix C. Preprocessing Pipeline and fMRI Analysis Details**

**Preprocessing Pipeline.** The T1-weighted (T1w) image was corrected for intensity non-uniformity (INU) with N4BiasFieldCorrection (Tustison et al., 2010), distributed with ANTs 2.2.0 (Avants et al., 2008, RRID:SCR_004757), and used as T1w-reference throughout the workflow. The T1w-reference was then skull-stripped with a Nipype implementation of the antsBrainExtraction.sh workflow (from ANTs), using OASIS30ANTs as target template. Brain tissue segmentation of cerebrospinal fluid (CSF), white-matter (WM) and gray-matter (GM) was performed on the brain-extracted T1w using fast (FSL 5.0.9, RRID:SCR_002823, Zhang et al., 2001). Volume-based spatial normalization to one standard space (MNI152NLin2009cAsym) was performed through nonlinear registration with antsRegistration (ANTs 2.2.0), using brain-extracted versions of both T1w reference and the T1w template. The following template was selected for spatial normalization: ICBM 152 Nonlinear Asymmetrical template version 2009c [Fonov et al., 2009; RRID:SCR_008796; TemplateFlow ID: MNI152NLin2009cAsym].

For each of the BOLD runs found per subject (across all tasks and sessions), the following preprocessing was performed. First, a reference volume and its skull-stripped version were generated using a custom methodology of fMRIPrep. The BOLD reference was then co-registered to the T1w reference using flirt (FSL 5.0.9, Jenkinson & Smith, 2001) with the boundary-based registration (Greve & Fischl, 2009) cost-function. Co-registration was configured with nine degrees of freedom to account for distortions remaining in the BOLD reference. Head-motion parameters with respect to the BOLD reference (transformation matrices, and six corresponding rotation and translation parameters) are estimated before any spatiotemporal filtering using mcflirt (FSL 5.0.9, Jenkinson et al., 2002). BOLD runs were slice-time corrected using 3dTshift from AFNI 20160207 (Cox & Hyde, 1997; RRID:SCR_005927). The BOLD time-series (including slice-timing correction when applied) were resampled onto their original, native space by applying a single, composite transform to correct for head-motion and susceptibility distortions. These resampled BOLD time-series will be referred to as preprocessed BOLD in original space, or just preprocessed BOLD. The BOLD time-series were resampled into standard space, generating a preprocessed BOLD run in [‘MNI152NLin2009cAsym’] space. First, a reference volume and its skull-stripped version were generated using a custom methodology of fMRIPrep. Several confounding time-series were calculated based on the preprocessed BOLD: framewise displacement (FD), DVARS and three region-wise global signals. FD and DVARS are calculated for each functional run, both using their implementations in Nipype (following the definitions by Power et al., 2014). The three global signals are extracted within the CSF, the WM, and the whole-brain masks. Additionally, a set of physiological regressors were extracted to allow for component-based noise correction (CompCor, Behzadi et al., 2007). Principal components are estimated after high-pass filtering the preprocessed BOLD time-series (using a discrete cosine filter with 128s cut-off) for the two CompCor variants: temporal (tCompCor) and anatomical (aCompCor). tCompCor components are then calculated from the top 5% variable voxels within a mask covering the subcortical regions. This subcortical mask is obtained by heavily eroding the brain mask, which ensures it does not include cortical GM regions. For aCompCor, components are calculated within the intersection of the aforementioned mask and the union of CSF and WM masks calculated in T1w space, after their projection to the native space of each functional run (using the inverse BOLD-to-T1w transformation). Components are also calculated separately within the WM and CSF masks. For each CompCor decomposition, the k components with the largest singular values are retained, such that the retained components’ time series are sufficient to explain 50 percent of variance across the nuisance mask (CSF, WM, combined, or temporal). The remaining components are dropped from consideration. The head-motion estimates calculated in the correction step were also placed within the corresponding confounds file. The confound time series derived from head motion estimates and global signals were expanded with the inclusion of temporal derivatives and quadratic terms for each (Satterthwaite et al., 2013). Frames that exceeded a threshold of 0.5 mm FD or 1.5 standardised DVARS were annotated as motion outliers. All resamplings can be performed with a single interpolation step by composing all the pertinent transformations (i.e. head-motion transform matrices, susceptibility distortion correction when available, and co-registrations to anatomical and output spaces). Gridded (volumetric) resamplings were performed using antsApplyTransforms (ANTs), configured with Lanczos interpolation to minimize the smoothing effects of other kernels (Lanczos, 1964). Non-gridded (surface) resamplings were performed using mri_vol2surf (FreeSurfer).

**fMRI Analysis Details.** MRI data were analyzed in SPM12 using a general linear model (GLM). Due to the jittered ITI, a two-gamma hemodynamic response function was used to model the expected BOLD signal for each trial per condition (Small, Large and Plus1 for arithmetic, Number, Shape and Face for matching, and VSWM Load 3, VSWM Load 5, Control Load 3 and Control Load 5 for VSWM). Only correctly solved trials were included for predictors of interest in the GLM. Error trials were coded as a separate predictor and included in analyses as a nuisance regressor. To avoid a decrease in data quality due to excessive motion during scanning, six motion parameters (x, y, z, roll, pitch and yaw), were included as nuisance regressors. An additional nuisance regressor that identified outlying volumes was also included. Outlying volumes were defined as volumes with volume-to-volume motion exceeding 1.5mm, or with a mean volume intensity of 4 *SD*’s or more beyond the z-normalized global signal across runs, as determined by Artifact Detection Tools implemented in the CONN toolbox ^79^. Participants for whom >20% of volumes for a task were marked as outliers were removed from further analyses of that specific task. This criterion led to the exclusion of 1 TA child in the matching task.

One main contrast of interest was specified for each task. For the arithmetic task, this was [(Large + Small) > Plus1], as it contrasts conditions in which participants were required to calculate with a condition in which calculation was not necessary to solve the problem (the Plus1 conditions can be solved by retrieving the subsequent number in the counting row). For the matching task, the contrast [Number > Shape] was calculated, as it taps into the matching of a number with its quantity, while controlling for processes of no interest such as visual perception and decision making. Finally, for VSWM, the contrast [VSWM (collapsed over load) > Control (collapsed over load)] was estimated, isolating activity related to holding visuo-spatial information in working memory. For each task, a whole-brain, within-subjects *t-*test including all participants (TA+DD) was run. An initial uncorrected threshold of *p* < .001 and a cluster level correction threshold of *p* < .05 calculated using the REST AlphaSim algorithm was applied using the updated autocorrelation function (“-acf” flag) to estimate noise smoothness values.

**Appendix C References**

Avants, B.B., C.L. Epstein, M. Grossman, and J.C. Gee. 2008. “Symmetric Diffeomorphic Image Registration with Cross-Correlation: Evaluating Automated Labeling of Elderly and Neurodegenerative Brain.” Medical Image Analysis 12 (1): 26–41. <https://doi.org/10.1016/j.media.2007.06.004>.

Behzadi, Yashar, Khaled Restom, Joy Liau, and Thomas T. Liu. 2007. “A Component Based Noise Correction Method (CompCor) for BOLD and Perfusion Based fMRI.” NeuroImage 37 (1): 90–101. https://doi.org/10.1016/j.neuroimage.2007.04.042.

Cox, Robert W., and James S. Hyde. 1997. “Software Tools for Analysis and Visualization of fMRI Data.” NMR in Biomedicine 10 (4-5): 171–78. https://doi.org/10.1002/(SICI)1099-1492(199706/08)10:4/5<171::AID-NBM453>3.0.CO;2-L.

Fonov, VS, AC Evans, RC McKinstry, CR Almli, and DL Collins. 2009. “Unbiased Nonlinear Average Age-Appropriate Brain Templates from Birth to Adulthood.” NeuroImage 47, Supplement 1: S102. <https://doi.org/10.1016/S1053-8119(09)70884-5>.

Greve, Douglas N, and Bruce Fischl. 2009. “Accurate and Robust Brain Image Alignment Using Boundary-Based Registration.” NeuroImage 48 (1): 63–72. <https://doi.org/10.1016/j.neuroimage.2009.06.060>.

Jenkinson, Mark, Peter Bannister, Michael Brady, and Stephen Smith. 2002. “Improved Optimization for the Robust and Accurate Linear Registration and Motion Correction of Brain Images.” NeuroImage 17 (2): 825–41. https://doi.org/10.1006/nimg.2002.1132.

Jenkinson, Mark, and Stephen Smith. 2001. “A Global Optimisation Method for Robust Affine Registration of Brain Images.” Medical Image Analysis 5 (2): 143–56. <https://doi.org/10.1016/S1361-8415(01)00036-6>.

Lanczos, C. 1964. “Evaluation of Noisy Data.” Journal of the Society for Industrial and Applied Mathematics Series B Numerical Analysis 1 (1): 76–85. <https://doi.org/10.1137/0701007>.

Ng EL, O’Brien BA, Khng KH, et al. Singapore kindergarten impact project (SKIP). *Office of*

*Educational Research Education Research Funding Programme# OER*. 2014;9:14.

Ng, E. L. and O’Brien, B. *Singapore Kindergarten Impact Project[Project 1440 Closure Report]*.; 2020.

Power, Jonathan D., Anish Mitra, Timothy O. Laumann, Abraham Z. Snyder, Bradley L. Schlaggar, and Steven E. Petersen. 2014. “Methods to Detect, Characterize, and Remove Motion Artifact in Resting State fMRI.” NeuroImage 84 (Supplement C): 320–41. <https://doi.org/10.1016/j.neuroimage.2013.08.048>.

Satterthwaite, Theodore D., Mark A. Elliott, Raphael T. Gerraty, Kosha Ruparel, James Loughead, Monica E. Calkins, Simon B. Eickhoff, et al. 2013. “An improved framework for confound regression and filtering for control of motion artifact in the preprocessing of resting-state functional connectivity data.” NeuroImage 64 (1): 240–56. https://doi.org/10.1016/j.neuroimage.2012.08.052.

Tustison, N. J., B. B. Avants, P. A. Cook, Y. Zheng, A. Egan, P. A. Yushkevich, and J. C. Gee. 2010. “N4ITK: Improved N3 Bias Correction.” IEEE Transactions on Medical Imaging 29 (6): 1310–20. <https://doi.org/10.1109/TMI.2010.2046908>.

Zhang, Y., M. Brady, and S. Smith. 2001. “Segmentation of Brain MR Images Through a Hidden Markov Random Field Model and the Expectation-Maximization Algorithm.” IEEE Transactions on Medical Imaging 20 (1): 45–57. https://doi.org/10.1109/42.906424.

**Appendix D. Trial list arithmetic task**

| **Condition** | **Arithmetic problem** | **Provided answer** | **Correct / Incorrect** |
| --- | --- | --- | --- |
| Small | 6 + 2 | 8 | Correct |
|  | 7 + 3 | 12 | Incorrect |
|  | 4 + 5 | 10 | Incorrect |
|  | 2 + 3 | 5 | Correct |
|  | 8 + 2 | 11 | Incorrect |
|  | 3 + 4 | 9 | Incorrect |
|  | 5 + 3 | 8 | Correct |
|  | 6 + 4 | 10 | Correct |
|  | 2 + 7 | 9 | Correct |
|  | 3 + 6 | 9 | Correct |
|  | 4 + 2 | 8 | Incorrect |
|  | 2 + 5 | 8 | Incorrect |
| Large | 8 + 6 | 14 | Correct |
|  | 5 + 8 | 13 | Correct |
|  | 5 + 6 | 13 | Incorrect |
|  | 3 + 9 | 13 | Incorrect |
|  | 7 + 5 | 12 | Correct |
|  | 9 + 4 | 14 | Incorrect |
|  | 8 + 3 | 13 | Incorrect |
|  | 7 + 4 | 11 | Correct |
|  | 6 + 7 | 14 | Incorrect |
|  | 9 + 5 | 16 | Incorrect |
|  | 2 + 9 | 11 | Correct |
|  | 4 + 8 | 12 | Correct |
| Plus 1 | 3 + 1 | 4 | Correct |
|  | 7 + 1 | 8 | Correct |
|  | 4 + 1 | 5 | Correct |
|  | 8 + 1 | 9 | Correct |
|  | 4 + 1 | 6 | Incorrect |
|  | 5 + 1 | 8 | Incorrect |
|  | 7 + 1 | 9 | Incorrect |
|  | 8 + 1 | 11 | Incorrect |
|  | 9 + 1 | 12 | Incorrect |
|  | 6 + 1 | 7 | Correct |
|  | 3 + 1 | 5 | Incorrect |
|  | 2 + 1 | 3 | Correct |

**Triallist matching task**

| **Condition** | **Left stimulus** | **Right stimulus** | **Match?** |
| --- | --- | --- | --- |
| Number | 4 dots | digit 4 | Yes |
|  | 6 dots | digit 6 | Yes |
|  | digit 5 | 1 dot | No |
|  | 4 dots | digit 8 | No |
|  | 2 dots | digit 2 | Yes |
|  | digit 1 | 1 dot | Yes |
|  | digit 9 | 9 dots | Yes |
|  | 2 dots | digit 5 | No |
|  | digit 3 | 5 dots | No |
|  | digit 5 | 5 dots | Yes |
|  | 6 dots | digit 3 | No |
|  | digit 7 | 7 dots | Yes |
|  | digit 9 | 7 dots | No |
|  | digit 7 | 4 dots | No |
|  | digit 3 | 3 dots | Yes |
|  | 8 dots | digit 5 | No |
|  | digit 1 | 4 dots | No |
|  | 8 dots | digit 8 | Yes |
| Shape | shape 8 | shape 8 | Yes |
|  | shape 3 | shape 3 | Yes |
|  | shape 2 | shape 3 | No |
|  | shape 2 | shape 2 | Yes |
|  | shape 6 | shape 6 | Yes |
|  | shape 9 | shape 1 | No |
|  | shape 7 | shape 7 | Yes |
|  | shape 1 | shape 2 | No |
|  | shape 5 | shape 5 | Yes |
|  | shape 8 | shape 9 | No |
|  | shape 5 | shape 6 | No |
|  | shape 9 | shape 9 | Yes |
|  | shape 7 | shape 8 | No |
|  | shape 4 | shape 5 | No |
|  | shape 3 | shape 4 | No |
|  | shape 6 | shape 7 | No |
|  | shape 1 | shape 1 | Yes |
|  | shape 4 | shape 4 | Yes |
| Face | Base face 1 | Small diff face 1 | No |
|  | Base face 9 | Base face 9 | Yes |
|  | Large diff face 6 | Base face 6 | No |
|  | Base face 4 | Small diff face 4 | No |
|  | Base face 5 | Base face 5 | Yes |
|  | Base face 2 | Base face 2 | Yes |
|  | Base face 7 | Base face 7 | Yes |
|  | Base face 3 | Base face 3 | Yes |
|  | Large diff face 7 | Base face 7 | No |
|  | Base face 6 | Base face 6 | Yes |
|  | Base face 8 | Base face 8 | Yes |
|  | Large diff face 9 | Base face 9 | No |
|  | Base face 3 | Small diff face 3 | No |
|  | Base face 4 | Base face 4 | Yes |
|  | Base face 5 | Small diff face 5 | No |
|  | Large diff face 8 | Base face 8 | No |
|  | Base face 1 | Base face 1 | Yes |
|  | Base face 2 | Small diff face 2 | No |

| **Table S1.** Significant Clusters for Whole Group Contrasts of Interest | | | |
| --- | --- | --- | --- |
| **Cluster size** | **MNI**  **x, y, z** | **Peak *t***  *minor maxima* | **Anatomical description** |
|  |  |  |  |
| **Arithmetic** [Large + Small] > Plus1 | | |  |
|  |  |  |  |
| **457** | **-19, -100, -1** | **6.55** | **L middle occipital lobe** |
|  | -12, -82, 4 | 5.02 |  |
|  | -39, -85, -13 | 4.66 |  |
| **1170** | **-32, -6, 47** | **6.11** | **L MFG & precentral gyrus** |
|  | -9, 14, 50 | 5.93 |  |
|  | -9, 29, 32 | 5.92 |  |
| **242** | **20, -95, 2** | **5.59** | **R occipital lobe, calcarine sulcus** |
|  | 35, -87, -8 | 5.12 |  |
| **168** | **28, 29, -1** | **5.51** | **R IFG & insula** |
|  | 33, 22, -6 | 5.06 |  |
| **619** | **-49, -45, 47** | **5.21** | **L IPL** |
|  | -32, -48, 37 | 4.82 |  |
|  | -34, -63, 54 | 4.74 |  |
| **161** | **-29, 26, 2** | **5.17** | **L insula** |
|  | -32, 19, -6 |  |  |
| **69** | **33, -1, 55** | **4.57** | **R MFG** |
| **188** | **-37, 4, 27** | **4.31** | **L IFG *orbitalis*** |
|  | -49, 9, 42 | 4.25 |  |
|  | -51, 9, 24 | 4.23 |  |
| **122** | **48, 36, 32** | **4.23** | **R MFG** |
|  | 38, 29, 30 | 3.92 |  |
| **31** | **20, 12, 62** | **4.11** | **R SFG** |
| **170** | **-34, 51, 15** | **4.06** | **L MFG** |
|  | -34, 56, 7 | 3.99 |  |
|  | -44, 44, 7 | 3.96 |  |
|  |  |  |  |
| **99** | **1, 19, -11** | **-4.34** | **L ACC** |
|  | 3, 4, -10 | 3.91 |  |
|  | -9, 24, -8 | 3.62 |  |
|  |  |  |  |
|  |  |  |  |
| **Matching** [Number > Shape] | | |  |
|  | | |  |
| **9499** | **33, -70, 32** | **13.07** | **Bilateral IPL, SPL & MOG** |
|  | -27, -65, 47 | 11.76 |  |
|  | 25, -92, 2 | 11.62 |  |
| **6532** | **-7, 24, 45** | **8.74** | **Bilateral MFG & SFG** |
|  | -39, 4, 34 | 8.27 |  |
|  | 6, 26, 44 | 8.17 |  |
| **239** | **-29, 24, -1** | **8.37** | **L insula** |
| **59** | **20, 44, -16** | **5.99** | **R SFG** |
|  | 22, 51, -13 | 4.30 |  |
|  | 25, 58, -8 | 3.54 |  |
| **131** | **6, -1, 30** | **5.96** | **R cingulate gyrus** |
|  | -4, 12, 24 | 5.60 |  |
|  | 6, 12, 24 | 4.93 |  |
| **336** | **6, -28, -3** | **5.34** | **R thalamus & hippocampus** |
|  | 22, -29, -3 | 5.03 |  |
|  | 5, -33, -18 | 5.00 |  |
| **134** | **10, -11, 5** | **5.30** | **R thalamus ventral lateral nucleus** |
|  | 10, -23, 12 | 4.23 |  |
|  | 20, -6, 4 | 4.02 |  |
|  |  |  |  |
| **190** | **43, -13, -3** | **-6.83** | **R insula** |
|  | 50, 2, -8 | -5.34 |  |
| **674** | **-7, 56, -6** | **-6.80** | **L MFG & ACC** |
|  | -4, 26, -8 | -6.59 |  |
|  | 6, 49, -13 | -5.51 |  |
| **139** | **-17, -8, -20** | **-6.44** | **L hippocampus & amygdala** |
|  | -24, 7, -16 | -3.81 |  |
| **339** | **-24, -50, 10** | **-6.23** | **L temporal lobe** |
|  | -19, -43, 17 | -5.74 |  |
|  | -14, -35, 20 | -5.31 |  |
| **269** | **60, -28, 27** | **-5.92** | **R IPL & SMG** |
|  | 67, -29, 19 | -4.79 |  |
|  | 47, -36, 27 | -4.56 |  |
| **206** | **-56, -28, 27** | **-5.43** | **L IPL & SMG** |
|  | -66, -33, 24 | -4.99 |  |
| **286** | **23, -45, 15** | **-5.37** | **R temporal lobe** |
|  | 33, -45, -1 | -5.36 |  |
|  | 10, -33, 17 | -4.94 |  |
| **182** | **-44, -60, 22** | **-5.30** | **L MTG & AG** |
| **44** | **-59, -3, -18** | **-5.28** | **L MTG** |
| **64** | **-7, -68, 22** | **-5.01** | **L precuneus** |
|  | -12, -58, 14 | -3.32 |  |
| **44** | **-39, 31, -13** | **-5.00** | **L IFG** |
|  | -30, 33, -8 | -3.85 |  |
| **47** | **-42, -13, 2** | **-4.94** | **L insula** |
|  | -49, -8, 0 | -4.02 |  |
| **35** | **-29, -43, 60** | **-4.54** | **L IPL & postcentral gyrus** |
| **39** | **-29, -33, -18** | **-4.41** | **L parahippocampal gyrus** |
| **77** | **-9, -45, 35** | **-4.40** | **L precuneus & cingulate gyrus** |
|  |  |  |  |
|  |  |  |  |
| **VSWM [**VSWM > Control] | | |  |
|  |  |  |  |
| **2399** | **38, -23, 55** | **11.78** | **R SPL & postcentral Gyrus** |
|  | 15, -68, 52 | 7.17 |  |
|  | 25, -1, 54 | 6.80 |  |
| **355** | **30, 26, -3** | **7.83** | **R IFG & insula** |
| **264** | **-32, 26, -1** | **7.27** | **L IFG & insula** |
|  | -37, 17, -8 | 5.75 |  |
|  | -32, 19, 10 | 5.27 |  |
| **255** | **-32, -6, 55** | **6.82** | **L MFG & precentral gyrus** |
| **947** | **-17, -68, 52** | **6.62** | **L SPL & precuneus** |
|  | -32, -50, 42 | 5.46 |  |
|  | -32, -58, -54 | 5.28 |  |
| **276** | **15, -23, 5** | **6.56** | **R thalamus** |
|  | 10, -21, 12 | 6.03 |  |
|  | -2, -26, -3 | 4.87 |  |
| **140** | **-17, -53, -23** | **6.47** | **L cerebellum** |
|  | -7, -63, -18 | 4.73 |  |
| **429** | **-9, 12, 55** | **6.41** | **L SFG & SMA** |
|  | -9, 17, 47 | 6.09 |  |
|  | 8, 29, 32 | 5.36 |  |
| **141** | **-46, 4, 30** | **5.23** | **L IFG & precentral gyrus** |
|  | -49, 2, 40 | 4.17 |  |
| **51** | **30, -8, -1** | **4.91** | **R Putamen** |
|  | 30, -1, 4 | 3.38 |  |
| **92** | **43, 4, 37** | **4.63** | **R MFG, IFG & precentral gyrus** |
|  | 58, 17, 27 | 3.57 |  |
| **104** | **40, 31, 22** | **4.59** | **R MFG** |
|  | 45, 34, 30 | 3.690 |  |
|  | 35, 36, 30 | 3.59 |  |
| **42** | **48, -18, 22** | **3.91** | **R insula** |
|  | 38, -23, 22 | 3.85 |  |
| **29** | **-29, -48, -8** | **3.80** | **L parahippocampal gyrus** |
|  |  |  |  |
| **2089** | **-39, -23, 52** | **-12.37** | **L pre- & postcentral gyrus** |
|  | -9, -26, 47 | -6.06 |  |
|  | 3, -87, 27 | -4.59 |  |
| **205** | **18, -55, -21** | **-7.02** | **R cerebellum** |
| **360** | **-22, 31, 52** | **-5.55** | **L SFG** |
|  | -17, 59, 32 | -5.21 |  |
|  | 3, 29, -20 | -4.79 |  |
| **47** | **-27, -1, -13** | **-5.28** | **L parahippocampal gyrus** |
|  | -29, -8, -3 | -3.36 |  |
| **247** | **-7, 66, 10** | **-5.26** | **L MFG & SFG** |
|  | -4, 31, -13 | -4.92 |  |
|  | 3, 29, -16 | -4.56 |  |
| **98** | **-37, 41, -16** | **-5.20** | **L MFG & IFG** |
|  | -44, 26, -13 | -4.73 |  |
|  | -34, 29, -20 | -4.35 |  |
| **184** | **-51, -23, 20** | **-4.48** | **L postcentral gyrus** |
|  | -61, -23, 20 | -4.39 |  |
|  | -36, -16, 14 | -4.31 |  |
| **173** | **-46, -70, 42** | **-4.34** | **L angular gyrus** |
|  | -49, -68, 32 | -4.08 |  |
|  | -37, -78, 44 | -3.97 |  |
| **67** | **-17, -8, -23** | **-4.26** | **L parahippocampal gyrus** |
|  | -19, -13, -10 | -4.06 |  |
| **42** | **18, 59, 27** | **-4.05** | **R SFG** |
|  | 15, 51, 40 | -3.71 |  |
|  | 20, 51, 32 | -3.49 |  |
|  |  |  |  |
| MFG = middle frontal gyrus; IFG = inferior frontal gyrus; IPL = inferior parietal lobule; SFG = superior frontal gyrus; ACC = anterior cingulate cortex; LGN = lateral geniculate nucleus of the thalamus; Table reports up to 3 local maxima more than 8.0 mm apart. | | | |

**Figure S1.** Maps of activation for between-subjects *t*-tests (TA > DD) for the matching task, main contrasts of interest (number > shape, blue) and exploratory contrast (number > fixation, orange). Significance threshold was *t* = 3.22 at *p* < .001 uncorrected, cluster-corrected at k = 29.

| **Table S2.** Significant Clusters for Between-Group Contrasts of Interest | | | | |
| --- | --- | --- | --- | --- |
|  | **Peak MNI  (x, y, z)** | **Cluster size** | **Peak *t*** | **Anatomical description** |
|  |  |  |  |  |
| **Matching: Number > Shape** |  |  |  |  |
|  |  |  |  |  |
| TA > DD | (20, -65, 67) | 62 | 4.98 | R SPL & precuneus |
|  | (-42, -23, 35) | 115 | 4.15 | L precentral gyrus |
|  | (-29, -68, 27) | 34 | 3.81 | L MOG |
|  |  |  |  |  |
| **Matching: Number > Fixation** |  |  |  |  |
|  |  |  |  |  |
| TA > DD | (-39, -10, 32) | 57 | 4.09 | L precentral gyrus |
|  | (35, 31, 34) | 37 | 4.15 | R middle frontal gyrus |
|  | (-46, 29, 34) | 45 | 4.14 | L middle frontal gyrus |
|  | (-42, -55, 62) | 34 | 3.81 | L SPL |
|  |  |  |  |  |
| *Note.* L = left, R = right, SPL = superior parietal lobule, MOG = middle occipital gyrus. | | | | |

**Figure S2.** Beta-weights extracted from ROIs showing significant between-group differences in pre-registered contrasts of interest (Number > Shape and Number > Face). To extract z-normalized beta weights, first-level contrasts of each condition were created (e.g. number vs baseline) and beta-weights for each condition were estimated for each condition. L MOG = left middle occipital gyrus; L Precentral = left precentral gyrus; R SPL = right superior parietal lobule. DD = developmental dyscalculia, TA = typically achieving.
